# Apparent dissociation between hemodynamic and neurochemical responses during chemogenetic modulation of cortical circuits in rats

**DOI:** 10.3389/fnins.2026.1916498

**Published:** 2026-10-08

**Authors:** Fatemeh Anvari-Vind, Nathalie Just

**Affiliations:** 1 Laboratory for Functional and Metabolic Imaging, Ecole Polytechnique Fédérale de Lausanne, Lausanne, Switzerland; 2 Danish Research Centre for Magnetic Resonance, Department of Radiology and Nuclear Medicine, Copenhagen University Hospital - Amager and Hvidovre, Copenhagen, Denmark

**Keywords:** BOLD, designer receptors exclusively activated by designer Drugs (DREADDs), fMRS, GABA, mismatch

## Abstract

**Introduction:**

Chemogenetic tools such as Designer Receptors Exclusively Activated by Designer Drugs (DREADDs) provide a powerful means to causally manipulate defined neuronal populations *in vivo*. While chemogenetic fMRI studies have consistently demonstrated robust hemodynamic responses following circuit perturbation, considerably less is known about the accompanying metabolic consequences. Functional magnetic resonance spectroscopy (fMRS) offers the potential to probe these neurochemical processes, yet the relationship between hemodynamic and metabolic responses remains poorly understood. Here, we combined chemogenetics, pharmacological fMRI (Ph-fMRI), and proton magnetic resonance spectroscopy (^1^H-MRS/fMRS) at 7 T to investigate the temporal evolution of metabolic and hemodynamic responses in the rat motor cortex.

**Methods:**

Female Fischer rats received viral injections in the motor cortex to express either a pan-neuronal hM3D(Gq) DREADD construct (hSyn-hM3D(Gq)) or an interneuron-targeted construct (hDlx-hM3D(Gq)). Ph-fMRI, fMRS, and ^1^H-MRS measurements were performed before, during, and following systemic administration of clozapine-N-oxide (CNO, 1 mg/kg). Functional MRS was acquired during the acute response phase (0–60 min post-injection), while conventional ^1^H-MRS measurements were obtained at a delayed time point (70 min post-injection).

**Results:**

Chemogenetic modulation produced robust and opposing hemodynamic responses. Pan-neuronal activation elicited focal positive BOLD responses (+3.5 ± 1.5%), whereas interneuron-targeted activation generated significant negative BOLD responses (−3.3 ± 0.8%). In contrast, acute fMRS measurements revealed no significant changes in Glx or GABA concentrations during the first hour following CNO administration, despite the presence of strong hemodynamic effects. However, delayed metabolic differences were detected at the static ^1^H-MRS time point acquired 70 min after CNO administration. Relative to Sham animals, the ipsilateral motor cortex of pan-neuronal DREADD animals showed higher GABA (+14.4%) and total choline compound concentrations (+57.8%), whereas interneuron-targeted DREADD animals exhibited lower Glx (−15.6%), total NAA (−16.9%), glucose (−25.9%), and total creatine (−25.4%). These delayed effects emerged after the period of peak hemodynamic responses and were not accompanied by significant acute fMRS changes.

**Conclusion:**

Chemogenetic perturbation of cortical circuits produced robust hemodynamic responses but more subtle and temporally complex metabolic effects. The absence of detectable acute changes in Glx and GABA despite strong BOLD responses, together with the emergence of delayed neurochemical alterations, highlights the challenges of interpreting metabolic signals in relation to circuit activity.

## 1 Introduction

Chemogenetic tools such as Designer Receptors Exclusively Activated by Designer Drugs (DREADDs) have revolutionized neuroscience by enabling cell-type–specific, reversible and minimally invasive modulation of neuronal activity. With DREADDs, genetically defined excitatory or inhibitory neuronal populations can be activated or silenced through systemic administration of an otherwise inert ligand (e.g., clozapine-N-oxide, CNO), thereby enabling causal interrogation of brain circuits and behaviour without the need for chronic implants, optical fibres, or electrodes (Giorgi et al., 2017; Ozawa and Arakawa, 2021).

In parallel, Blood Oxygen Level-dependent (BOLD) functional magnetic resonance imaging (fMRI) has emerged as a useful tool monitor the hemodynamic consequences of such manipulations across distributed networks. Many preclinical chemogenetic studies, especially in rodents, have employed 7-Tesla (7 T) MRI, leveraging established DREADD-fMRI protocols (Markicevic et al., 2020; Rocchi et al., 2022). For instance, in the seminal Ph-fMRI study activating serotonergic neurons, region-specific BOLD responses were reliably detected following DREADD stimulation (Giorgi et al., 2017). Similarly, inhibitory DREADD manipulations (e.g., targeting dopaminergic neurons) have been shown to modulate BOLD responses to external stimulation, confirming that hemodynamic readouts track network-level effects of chemogenetic perturbations (Helbing et al., 2024).

These achievements have encouraged the view that DREADD-fMRI at 7 T is a robust platform for mapping functional connectivity and circuit modulation *in vivo*. However, BOLD is only an indirect marker of neuronal activity (Gauthier and Fan, 2019; Uludağ, 2023). It reflects neurovascular coupling and hemodynamic changes, not the fundamental neurochemical alterations (e.g., in excitatory/inhibitory neurotransmitter pools) that drive synaptic transmission and metabolic demand. Thus, without complementary measures, BOLD fMRI alone cannot reveal whether activation or inhibition via DREADDs actually alters the balance of glutamate, γ-aminobutyric acid (GABA), or other metabolic substrates in a region.

Proton magnetic resonance spectroscopy (^1^H-MRS), in contrast, offers a non-invasive means of quantifying neurometabolites such as glutamate (Glu) and GABA *in vivo*. Under appropriate acquisition parameters, ^1^H-MRS can yield measures of regional concentrations of these key neurotransmitters and neurochemicals, arguably offering a more direct window into excitatory-inhibitory balance than hemodynamics alone. Functional MRS (^1^H-fMRS), which tracks temporal changes in metabolite concentrations during stimulation or experimental manipulation, holds promise for revealing the metabolic correlates of neural activation (Just and Faber, 2019; Just, 2021). Recent human and animal fMRS studies have reported small but significant changes in Glu or Glx (Glu + Gln) during sensory or cognitive tasks, suggesting that neurotransmitter pools can shift measurably in response to physiological stimulation (Bednařík et al., 2015; Craven et al., 2024; Pasanta et al., 2023). Moreover, an elegant study combining fMRS with two-photon calcium imaging in awake mice found that MRS-derived changes in Glu and GABA tracked documented shifts in excitatory and inhibitory neuronal activity, respectively, bolstering the notion that MRS can reflect functional neurotransmission rather than merely baseline metabolic pools (Takado et al., 2022).

Despite this promise, relatively few studies have combined chemogenetics (DREADDs) with functional magnetic resonance spectroscopy (fMRS), particularly in preclinical models. While chemogenetic-fMRI studies have consistently demonstrated robust hemodynamic responses following selective circuit perturbation (Markicevic et al., 2020; Rocchi et al., 2022), considerably less is known about the accompanying metabolic consequences. Existing chemo-fMRS investigations are scarce, and how targeted neuronal modulation influences metabolic responses and their coupling to hemodynamic signals is still not well characterized. Moreover, recent meta-analyses of ^1^H-fMRS studies have highlighted substantial variability across paradigms, generally modest effect sizes for Glu/Glx, and often inconsistent GABA responses, emphasizing the influence of stimulus design, brain region, acquisition strategy, and temporal resolution on metabolite detectability (Pasanta et al., 2023).

Importantly, these observations raise a broader question that extends beyond methodological sensitivity: what does a metabolic signal actually reveal about neural circuit function? While BOLD fMRI provides an indirect measure of activity through vascular responses, fMRS offers the possibility of probing the neurochemical processes that support neuronal signaling. However, the precise relationship between hemodynamic and metabolic responses remains incompletely understood. Detectable changes in Glu, Gln, GABA concentrations, or related metabolites may reflect alterations in neurotransmitter cycling, cellular energetics, glial-neuronal interactions, or network reorganization. But their interpretation in terms of circuit activity is still debated. Chemogenetic approaches provide a unique opportunity to address this question. By selectively modulating defined neuronal populations, DREADDs allow causal perturbation of neural circuits while simultaneously monitoring their hemodynamic and metabolic consequences. Combining chemogenetics with fMRI and fMRS therefore offers a powerful framework for investigating how circuit-level manipulations are translated into vascular and metabolic responses *in vivo* and for probing the relationship between neurovascular and neurometabolic coupling.

In the present study, we applied this multimodal approach to the rat motor cortex, where robust chemogenetic fMRI responses have been consistently reported at 7 T.

Two complementary chemogenetic approaches were employed. First, a pan-neuronal hSyn-hM3D(Gq) construct was used to activate the local neuronal population. Second, an hDlx-hM3D(Gq) construct was used to selectively activate GABAergic interneurons, thereby increasing inhibitory tone within the same cortical network. These complementary perturbations enabled us to investigate how distinct cellular drivers of circuit activity are reflected in hemodynamic and metabolic measurements. Using concurrent assessment of BOLD responses, interhemispheric communication, and metabolic dynamics with ^1^H-fMRS, we sought to determine how metabolic signals relate to causal manipulations of cortical circuitry. More broadly, we asked whether hemodynamic and metabolic measurements provide complementary or divergent information regarding neural circuit function. Finally, computational simulations at higher magnetic field strengths were performed to explore the detectability of plausible neurometabolic changes under improved spectral resolution and signal-to-noise conditions. Together, these experiments provide a framework for interpreting metabolic measurements in the context of circuit-level perturbations and highlight both the opportunities and challenges of combining chemogenetics, fMRI and fMRS.

## 2 Materials and methods

### 2.1 Animals

Female Fisher rats (Janvier, France; BW = 190 ± 20 g) were used in the present study. Rats were housed in pairs in the Danish Research Centre for Magnetic resonance (DRCMR) animal facility with controlled temperature (18–22°C) and humidity (50%–56%) and free access to food and water under a 12-h reverse light-dark cycle (light off at 19:00). Two weeks after their arrival within the facility, rats were transported to the genetically modified (GMO) laboratory for stereotactic viral injections.

For general activation of cortical neurons within motor cortical areas (M_1,2_), we used the pAAV8-hSyn-hM3D(Gq)-mCherry construct. The pAAV8-hSyn-hM3D(Gq)-mCherry vector enables robust pan-neuronal expression of hM3D(Gq), supporting effective DREADD activation. While the hSyn promoter drives expression specifically in neurons, it does not distinguish between excitatory and inhibitory subtypes, potentially complicating interpretations in heterogeneous cortical regions. This limits our ability to dissect cell-type-specific contributions. Nonetheless, this broad activation was appropriate for our primary objective of validating chemo-fMRS as a tool for studying cortical neurochemical dynamics. Selective promoters may be used in future studies to dissect cell-type-specific contributions. To induce inhibitory modulation of cortical circuits, we used the pAAV-hDlx-Gq-DREADD-dTomato-Fishell-4 construct, which drives expression of the hM3D(Gq) receptor selectively in GABAergic interneurons (Dimidschstein et al., 2016). Upon CNO administration, activation of these interneurons is expected to increase inhibitory tone thereby reducing the activity of neighboring excitatory neurons within the local cortical network. These AAVs were produced with a retrograde serotype, which permits retrograde access to projection neurons. For the sham group, animals underwent identical stereotaxic procedures and received injections of the AAV5-CaMKIIa-hChR2(H134R)-EYFP construct.

This construct was used as a viral expression control because it enables stable neuronal transgene and fluorescent protein expression without chemogenetic activation. Thus, it controlled for potential effects of surgery, viral transduction, and long-term transgene expression, while lacking the capacity for CNO-induced neuronal modulation. Control rats (CTL), with or without viral transduction, received either saline (0.9%) or Clozapine-N-Oxide (CNO) to account for physiological, pharmacological, and neurochemical effects.

For surgery, rats were anesthetized with isoflurane (typically 2%, adjusted as needed) and placed in a stereotaxic frame. Preoperative analgesic, antibiotic, and anti-inflammatory treatments were administered. Viral injections targeted the right primary motor cortex (M1/M2; AP: 0–1 mm, ML: 2.0–3.2 mm from bregma). Following a small craniotomy, 1 μl of viral construct was infused 1 mm below the cortical surface at 0.2 μl/min. Animals received postoperative care and were housed for at least 4 weeks to allow for transgene expression. A total of 76 Fisher rats were included in the study (Table 1).

**TABLE 1:** Number of animals in different groups and for histology/Immunohistochemistry.

Group	CTL (+Saline)	Sham	pAAV8-hsyn-hM3D(Gq)_mcherry	pAAV-hdlx-Gq-DREADD-dTomato_Fishell4	Histology/IHC
			(eDREADDs)	(iDREADDs)	
Initial	10	15	16	16	43
Measured	8 (Ph-MRI+fMRS)	5 (Ph-MRI)	6 (Ph-MRI)	6 (Ph-MRI)	29 (12 Fluorescence/17 cFos)**
		6 (fMRS)	8 (fMRS)	8 (fMRS)	
Failures	2^§^	4^§§^	2^¥^	2^¥¥^	NA

Initially, we obtained 100 rats that we distributed amongst groups of 16/ 10 for CTL. ^§^ 2 rats died during preparation 1 in the cradle/1 upon medetomidine injection. ^§§^ Sham rats were in general difficult to anesthetize. 2 rats were sacrificed before MRI due to respiratory difficulties. 1 rat was sacrificed for bleedings and 1 rat was injected at wrong coordinates. ¥ 2 eDREADDs were removed from analysis due to Motion and wrong coordinate injections. ¥¥ 2 Ph-MRI showed important drift issues and were removed from analysis. **We used a high number of rats for histology to test various factors including the dose of CNO, appropriate staining and IHC.

### 2.2 MRI acquisition

All *in vivo* MRI experiments were conducted on a 7 T Bruker BioSpec 70/20 scanner (Bruker, Wissembourg, France) equipped with an 86-mm volume transmit coil and a 20-mm single-loop surface receive coil (Bruker, Ettlingen, Germany).

Anesthesia was induced with 5% isoflurane and maintained during preparation with 2%–3% isoflurane in air/oxygen (70:30%) delivered via a nose cone. An intraperitoneal cannula was inserted for subsequent administration of Clozapine-N-oxide (CNO; 1 mg/kg). Animals were positioned supine in a stereotaxic frame with a bite bar and ear bars, and ophthalmic ointment was applied to prevent corneal drying.

Physiological monitoring included continuous measurement of rectal temperature (37 ± 0.5°C), respiration using a pneumatic sensor (SA Instruments, NY, USA), and blood oxygen saturation (SpO_2_) and heart rate (HR) using a pulse oximeter attached to the left hind paw (Nonin Medical B.V., The Netherlands). Signals were recorded using in-house Python scripts.

After stabilization of physiological parameters, medetomidine (0.05 mg/kg; DexDomitor, Orion Corporation, Finland) was administered subcutaneously. Following the expected reduction in respiratory and heart rates, isoflurane was decreased to 0.25%, and continuous medetomidine infusion (0.1 mg/kg/h) was initiated 10 min later. Physiological parameters were monitored throughout all MRI experiments. SpO_2_ was maintained between 92% and 100% in all animals.

Medetomidine bolus administration induced an initial reduction in HR, followed by variable recovery profiles across animals. These patterns were observed in DREADD, sham, and control (CTL) groups. Notably, only DREADD animals exhibited additional HR fluctuations following CNO administration, suggesting an effect of chemogenetic activation on autonomic regulation. Because interhemispheric mechanisms were also of interest, experiments were performed without intubation (Just and Hoehn, 2025).

The outline of the experiments is described in Figure 1. First, structural T_2_-weighted TurboRARE images covering the rat brain were acquired (TR/TE = 2700/33 ms; RARE factor = 8; Matrix = 256 × 256; FOV = 25 × 25 mm; number of slices = 25; Slice Thickness = 0.8 mm). One hour after medetomidine induction, Ph-fMRI scans were acquired using a gradient-echo echoplanar imaging (GRE-EPI) sequence aligned to the previously acquired structural images (TR/TE/flip angle = 1000/11 ms/60°; Matrix = 64 × 64; FOV = 25 × 25 mm; repetitions: 315; Slice thickness = 0.8 mm). Ph-fMRI was conducted with 3000 repetitions (total acquisition time = 59 min). The bolus of CNO was administered manually 15 min after the start of the EPI acquisition.

**FIGURE 1:**
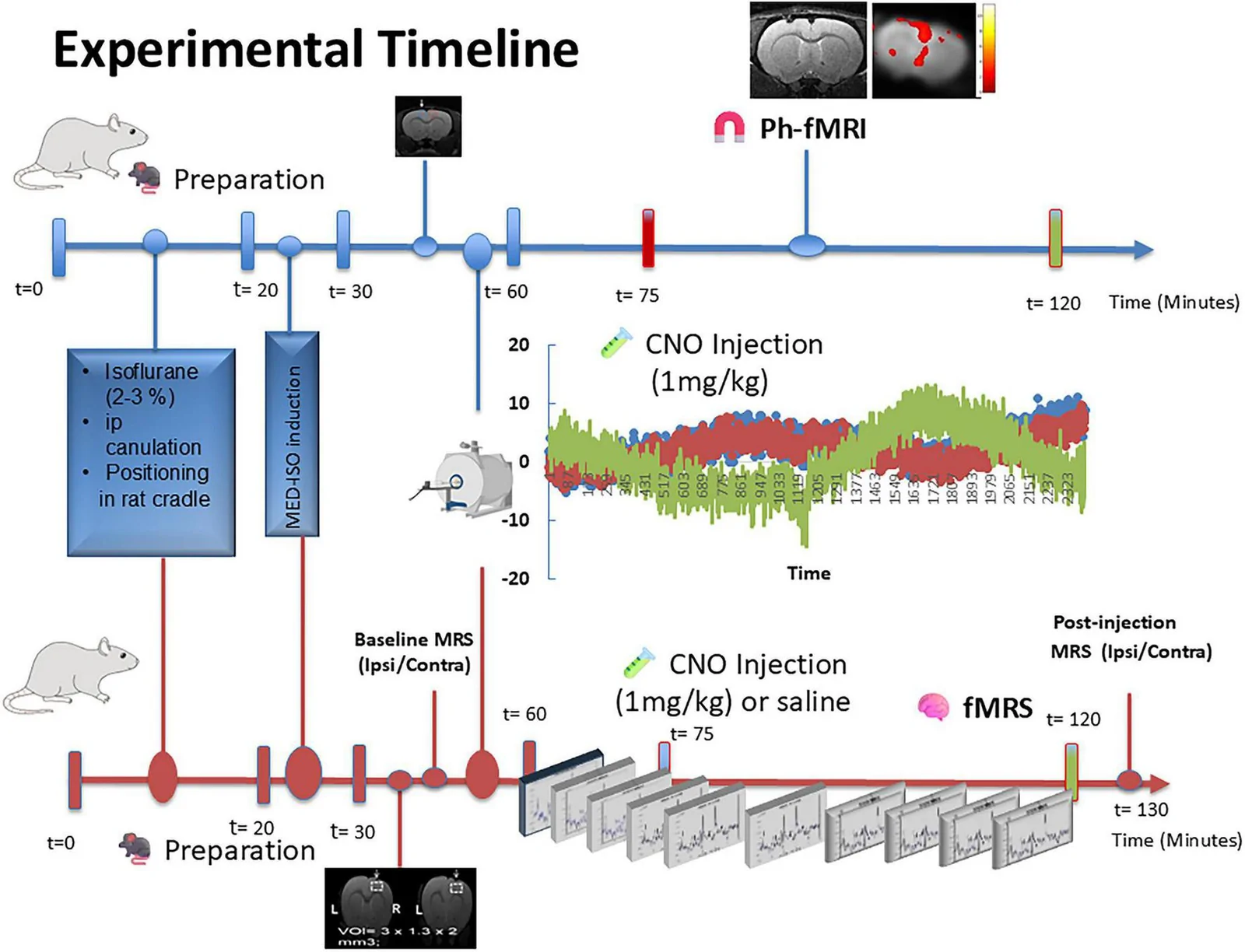
Timelines summarizing anesthesia (ISO→MED-ISO), baseline, Ph-fMRI (1) with CNO 1 mg/kg at *t* = 15 min, dynamic ^1^H-fMRS (0–60 min, 2), and delayed static ^1^H-MRS (∼70 min post-CNO) acquired ipsilateral and contralateral to the injection site.

### 2.3 Image processing

#### 2.3.1 Preprocessing of fMRI series

All MR images were first exported from ParaVision 6.0.1 to DICOM format and then converted to NIfTI (Neuroimaging Informatics Technology Initiative)^1^ using the dcm2nii tool.^2^ The structural image of one of the rats was chosen as the target reference space and co-registered to the SIGMA rat brain template (Barrière et al., 2019). Every other structural image was registered to the target using statistical parametric mapping (SPM12) and to the SIGMA rat template. The fMRI image series were preprocessed using functions from the SPM12 and FSL (FMRIB Software Library)^3^ neuroimaging toolkits.

Images were slice-time corrected, realigned, co-registered, normalized to a structural template, and spatially smoothed with a 1 mm FWHM 3D Gaussian kernel using SPM12 (MathWorks, Natick, MA, USA)^4^.

#### 2.3.2 Mapping BOLD responses

For Ph-fMRI processing, in-house MATLAB routines were used. An ROI-based analysis was conducted to extract dynamic GRE-EPI signal intensity time courses. ROIs were drawn over the chemo-induced area within the MC (M1,2) of each rat brain and its symmetrical homotopic area. Additional ROIs were defined in the somatosensory cortices and subcortical areas. In addition, we proceeded as described in Klomp et al. (2012), Schrantee et al. (2017) to obtain BOLD maps during CNO challenge. Briefly, whole-brain analyses were conducted to extract signal-intensity time series. Voxels showing more than 1% signal-intensity change were identified using a *t*-test. The average signal time course of activated voxels was determined for each animal, and individual time courses were then averaged to define the shape of the activation waveform. This waveform was used to set up a general linear model (GLM) in SPM12, with 1.2 mm FWHM spatial smoothing and a high-pass filter of 3600 s. As in previous Ph-fMRI studies (Klomp et al., 2012; Schrantee et al., 2017), this data-driven waveform was used to approximate the slow pharmacological response to CNO. We acknowledge that deriving the model from the same dataset introduces a degree of circularity and therefore interpret the resulting statistical maps primarily as descriptive rather than strictly independent inferential measures. The first volume was discarded to ensure that steady-state imaging had been reached The design matrix consisted of a custom waveform with an initial 900 s baseline period, followed by a 900 s gradual onset period beginning at the time of CNO injection, and a final 1800 s steady-state activation period. Image acquisition was performed using a seven-slice GRE-EPI sequence.

This custom block design was constructed to model the temporal profile of the CNO-induced response and included six motion parameters (translations and rotations) as nuisance regressors. The gradual onset period was modeled using a ramp function convolved with SPM’s canonical hemodynamic response function (HRF), while high-pass filtering (cutoff = 3600 s) was applied to remove low-frequency drifts.

Statistical inference was performed at the single-subject (first-level) level using t-contrasts comparing the activation period with baseline. T-statistical maps were corrected for multiple comparisons using family-wise error (FWE) correction at *p* < 0.05.

### 2.4 ^1^H-fMRS and static ^1^H-MRS pre and post- CNO

#### 2.4.1 Functional proton magnetic resonance spectroscopy (^1^H-fMRS)

Localized functional proton MR spectroscopy (^1^H-fMRS) was conducted using a LASER sequence (TR = 2 s; TE = 24.5 ms, Spectral width = 4000 Hz; 2048 points) modified from a sequence generously shared by Dr Julien Valette (Commissariat à l’Energie Atomique, Paris-Saclay; France). Prior to ^1^H-fMRS acquisitions, we acquired B_0_ field map and water spectrum, adjusted basic frequency, shimmed globally and locally, and optimized RF power. A large voxel of interest (VOI = 1.4mm × 2.8 mm × 2 mm) was positioned onto the anatomical T_2_-weighted images over the viral injection site covering the entire motor cortex (MC) encompassing M1 and M2 areas. After shimming using MAPSHIM and water suppression with VAPOR (Tkác et al., 1999), 200 fids were acquired continuously (with 8 or 16 averages, 16 s or 32 s per time point) using the LASER sequence. After 15 min, CNO (1mg/kg) or saline was injected, and fids were acquired up to 59 min. For quantification purposes, a water spectrum was also acquired prior to each dynamic acquisition (8 fids). MAPSHIM was used for shimming over the VOI down to a linewidth of 10.7 ± 2 Hz.

Pre and Post- CNO ^1^H-MRS were acquired successively in the ipsilateral and contralateral homotopic MC within a voxel of identical size following re-shimming. The LASER sequence was used with similar acquisition parameters to ^1^H-fMRS and 512 averages.

#### 2.4.2 ^1^H-fMRS data processing

^1^H- fMRS and ^1^H- MRS data were processed using in-house written MATLAB routines adapted from FID-A (Simpson et al., 2017). After Fourier transformation, individual spectra were zeroth-order phase adjusted and re-aligned. After visual inspection and removal of phase outliers, spectra were first order phase adjusted and finally averaged. Metabolic time courses were obtained by averaging spectra over 15 min per successive time -period (baseline, transition, or activation) or 5 min per time point to obtain full time courses. Each point was transferred to LCModel (Provencher, 2001).^5^ The quantification was performed with a basis-set of 20 brain metabolite spectra simulated using a density matrix procedure with FID-A (Simpson et al., 2017): Scyllo-Inositol (Scyllo); Ascorbate (Asc); Alanine (Ala); Aspartate (Asp); Glycerophosphocholine (GPC); Phosphocholine (PCh); Creatine (Cr); Phosphocreatine (PCr); γ-amino-butyric acid (GABA); Glucose (Glc); Glutamine (Gln);Glutamate (Glu); myo-Inositol (Ins); Lactate (Lac); N-Acetyl-Aspartate (NAA); N-acetylaspartyl-glutamic acid (NAAG); Phosphatidylethanolamine (PE); Taurine (Tau); total creatine (tCr: PCr + Cr, total NAA (tNAA = NAA + NAAG) and Glx = Glu + Gln were also assessed.

A spectrum of fast-relaxing macromolecules was also included in the basis set: Macromolecules (Mac). This macromolecule spectrum was measured over a 4 mm × 4mm × 4 mm brain VOI in six healthy Fisher rats with the double-inversion procedure included in the LASER sequence package with a TR = 8s. Absolute metabolite concentrations were obtained using unsuppressed water signal (eight averages) as an internal reference, assuming a brain water content of 80%. The Cramer-Rao lower bounds (CRLB) were used as a reliability measure of metabolite concentration estimates. Higher concentration metabolites (Glu, NAA, Ins, Taurine, PCr and Cr) with CRLB under 5% were considered reliably quantified whereas for Glc, GABA, Lac, and Asp, CRLB under 40% were considered acceptable and data were kept for further analysis. All data including those above the defined threshold were displayed. To determine the quality of spectra, NAA and tCr (PCr + Cr) spectral linewidths and Signal to noise ratio (SNR) were also assessed. The spectral SNR was calculated as the ratio between the NAA peak amplitude and the standard deviation of spectral noise measured between 5 and 8 ppm.

#### 2.4.3 Metabolite concentration time courses

In-house written MATLAB routines were used. Individual fids were Fourier transformed. 20 individual spectra, representing 5 min of acquisition, were realigned, phased, and averaged, allowing us to obtain 10 successive time points per metabolite. 5-min averaged spectra were transferred to LCModel and adjusted for each animal. Mean metabolite time courses were obtained by averaging metabolite concentrations across animals for eDREADDs, iDREADDs, Sham and controls. The spectral SNR was calculated as the ratio between the NAA peak amplitude and the standard deviation of spectral noise measured between 5 and 8 ppm.

In order to further explore the impact of DREADD activation or inhibition in both hemispheres of the brain, successive ^1^H -MR Spectra were further acquired in the same chemo-induced ipsilateral VOI used for chemo-fMRS and in its homotopic contralateral part, prior to CNO injection and 70-min post-CNO injection. This timepoint was chosen because systemic and brain concentrations of CNO become measurable within 15–30 min after systemic administration and downstream neurometabolic effects detectable by MRS typically evolve over tens of minutes; performing MRS at ∼70 min therefore balances early receptor engagement with the time required for metabolic shifts to become measurable while minimizing later systemic/off-target effects (for example clozapine back-conversion) reported at later post-injection intervals. Ipsilateral vs. contralateral comparisons were used as within-subject controls; Sham animals were included to control for systemic and off-target effects.

### 2.5 Histology and c-Fos immunohistochemistry

To verify viral expression and anatomical specificity of the DREADD injections, dedicated animals from each group were used for histological analysis. Animals were transcardially perfused with phosphate-buffered saline (PBS, 0.01 M), followed by 4% paraformaldehyde (PFA). Brains were post-fixed in 4% PFA, cryoprotected in 30% sucrose, and sectioned coronally (40–50 μm) using a cryostat (Leica CM3050 S). Sections were mounted on gelatin-coated slides and cover slipped with ProLong Gold mounting medium. Fluorescent reporter expression was assessed using epifluorescence microscopy, and representative sections covering the full extent of the injection site were digitized using a Zeiss Axioscan Z1 slide scanner. In a subset of animals expressing excitatory DREADDs, neuronal specificity was further confirmed by immunostaining for NeuN using standard immunohistochemical procedures.

For assessment of neuronal activation, c-Fos immunohistochemistry was performed in a cohort of Fischer rats following chemogenetic stimulation. After a minimum of 4 weeks to allow for viral expression, animals received systemic CNO injections under light isoflurane anesthesia and were perfused approximately 2 h later. Only animals receiving 1 mg/kg CNO are reported here. Free-floating coronal sections were processed using standard blocking and antibody incubation protocols, with mouse anti-c-Fos primary antibody and Alexa Fluor–conjugated secondary antibody. Sections were imaged using a Zeiss LSM 880 confocal microscope, acquiring z-stacks at 1 μm resolution.

The hDlx enhancer is a well-established tool for the selective targeting of forebrain GABAergic interneurons. hDlx-driven hM3D(Gq) expression is restricted to interneurons, and CNO administration selectively increases their activity, thereby enhancing inhibitory drive onto neighboring excitatory neurons (Dimidschstein et al., 2016).

To provide additional confirmation of DREADD-mediated neuronal activation, c-Fos-positive cells were quantified in a limited subset of representative animals for which confocal datasets were available. Cell counts were performed bilaterally within the motor cortex and expressed as c-Fos-positive cells per mm^2^ using ImageJ (NIH) in predefined regions of interest, including ipsilateral and contralateral motor cortex. Given the exploratory nature of this analysis and the limited sample size, results were used for qualitative comparison only.

### 2.6 Biophysical simulation of metabolite dynamics at 7T and 14T (*see simulated detectability of chemogenetic metabolic*…)

To interpret the experimental ^1^H-fMRS findings and assess field-strength–dependent detectability, we implemented a biophysical simulation framework (Python) to generate synthetic ^1^H-fMRS time courses at 7T and 14T. The simulation framework was not preregistered and was developed following completion of the experimental analyses to aid interpretation of the fMRS findings. Parameters describing acquisition conditions (field strength, spectral resolution, and signal-to-noise ratio) were based on experimentally measured values and published literature (Emir et al., 2011; Mlynárik et al., 2008; Near et al., 2015; Tkác et al., 2009), whereas the amplitudes and temporal profiles of metabolite changes were model assumptions chosen to represent biologically plausible chemogenetic perturbations. The simulations were therefore intended as a hypothesis-generating tool to explore detectability limits at different field strengths rather than as a formal validation of the experimental data.

The model combined (i) a pharmacokinetic component describing systemic ligand dynamics and (ii) a compartmental neurometabolic system representing excitatory neurons, inhibitory neurons, and astrocytes, inspired by established glutamate–glutamine cycle and neuroenergetic models (Gruetter, 2002; Hyder et al., 2006; Rae, 2014; Tkác et al., 2009). Chemogenetic effects were implemented as fractional modulations of compartment-specific metabolite pools driven by ligand concentration through a Hill-type relationship, analogous to receptor–effector coupling models commonly used in systems pharmacology (Gabrielsson et al., 2016). Identical underlying biological perturbations were imposed at both field strengths, while field-dependent observability was modeled by incorporating realistic differences in spectral resolution and signal-to-noise ratio. At 7T, metabolite pooling was enforced (Glx = Glu + Gln) and higher noise levels were applied, consistent with preclinical fMRS limitations (Near et al., 2015; Tkác et al., 2009), whereas at 14T individual Glu, Gln, and GABA concentration time courses were retained with improved SNR and reduced variance (Emir et al., 2011; Mlynárik et al., 2008). Inter-subject variability was simulated by randomizing key physiological parameters across virtual subjects. This framework allows direct comparison of the temporal detectability of chemogenetically induced metabolic changes at 7T versus 14T under otherwise equivalent biological conditions. Importantly, the ligand-driven component can be parameterized either to reflect acute CNO-driven activation or to reproduce delayed profiles associated with CNO–clozapine conversion, enabling interpretation of both early null effects and late metabolic changes observed experimentally.

### 2.7 Statistical analysis

Statistical analyses were performed using R (version 4.5.2) and the lme4, lmerTest, emmeans, and effect size packages or in Prism 8 (GraphPad, San Diego, CA). Data are presented as mean ± standard deviation (SD) unless otherwise stated. BOLD responses and static metabolite concentrations were compared across groups using a repeated-measures ANOVA followed by a Bonferroni *post hoc* test. For repeated-measures ANOVAs, sphericity was assessed using Mauchly’s test. When the sphericity assumption was violated, Greenhouse-Geisser corrections were applied. *Post hoc* comparisons were adjusted using Bonferroni correction. For analyses involving multiple metabolites, Bonferroni correction was applied separately within each family of planned comparisons. The number of comparisons in each family is indicated in the corresponding tables.

For each group, mean BOLD responses in contralateral and ipsilateral sides were extracted at each time point. The contra–ipsi difference was computed as diff = mean_contra - mean_ipsi.

To compare the temporal evolution of contra–ipsi differences across groups, we fitted a weighted linear model: *diff*∼*group*×*time*. This model allowed testing: (1) Contra–ipsi difference within each group (intercept at time 0), (2) Differences between groups at time, and (3) Group-specific temporal changes (interaction group × time). Significance was assessed at α = 0.05.

Temporal patterns were visualized using model predictions and their associated standard errors.

A linear mixed-effects model was fitted to assess the effects of Group (eDREADD, iDREADD, Sham, CTLs), Hemisphere (ipsilateral vs. contralateral), and Time Point on the measured metabolite concentration time courses. Fixed effects included Group, Hemisphere, TimePoint, and their interactions, while Subject was modelled as a random intercept to account for repeated measures. Model assumptions were verified via residual diagnostics. Estimated marginal means and pairwise contrasts were computed using the emmeans package with Tukey adjustment for multiple comparisons. Confidence intervals for fixed effects were obtained via profile likelihood, and standardized effect sizes (Cohen’s *d*, partial R^2^) were calculated using the effect size package. A *p* < 0.05 was considered significant.

## 3 Results

### 3.1 CNO induced significant BOLD responses in the chemo-induced motor cortex

Representative c-Fos quantification in a subset of animals revealed increased ipsilateral neuronal activation following chemogenetic stimulation (Figures 2A,B).

**FIGURE 2:**
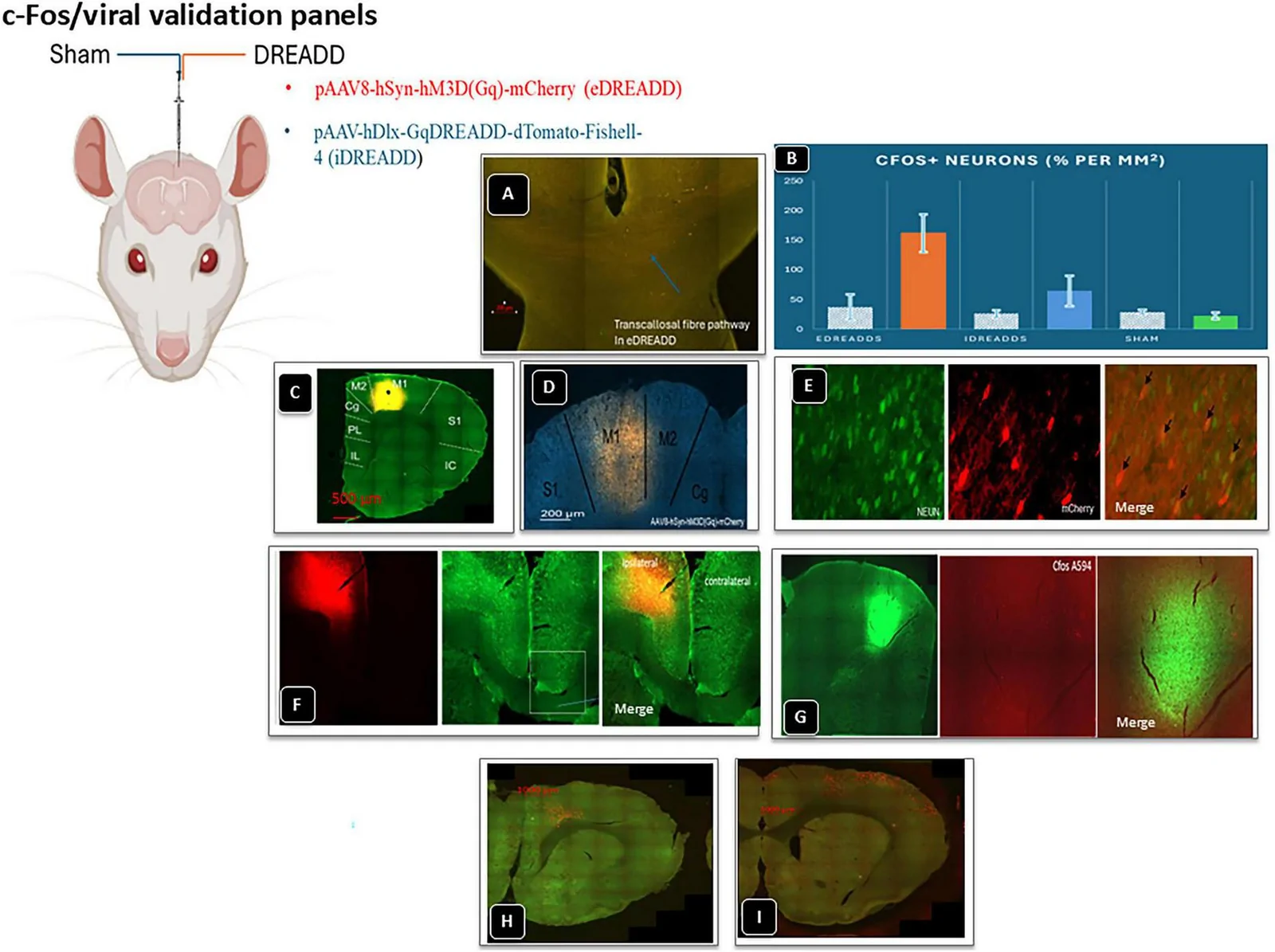
c-Fos/viral validation (M1 only). **(A)** Schematic/example of the transcallosal connection between homotopic MC regions for anatomical context. **(B)** Representative c-Fos labeling densities for a subset of rats of each group comparing contralateral versus ipsilateral sides (eDREADDs ipsilateral side: orange; iDREADDs ipsilateral side: blue; sham ipsilateral side: green). **(C)** Reporter fluorescence confirms viral expression restricted to M1 (AP 0–1 mm; ML 2.0–3.2 mm; depth ∼1 mm) **(D)** representative fluorescence images from motor cortex M1 for eDREADDs (*n* = 12); **(E)** Staining for the neuronal marker NEUN and m-cherry indicated the proportion of cells infected with the AAV8-hSyn-hM3D(Gq)-mCherry virus and merging of the two (arrows indicate double-labelled cells [cells that co-express G(q)-DREADDS and NEUN) **(F)** Cfos expression in contralateral and ipsilateral rat cortex, and **(G)** Sham animals, here provided solely to verify targeting/activation; not a primary outcome. **(H,I)** Representative and qualitative cFos staining for pAAV-hDlx-Gq-DREADD-dTomato-Fishell-4 construct (iDREADDs) in the rat cortical areas.

Animals expressing the pan-neuronal hSyn-hM3D(Gq) construct exhibited the largest increase in c-Fos-positive cells within the injected motor cortex compared with the contralateral hemisphere (Figures 2C–F).

Sham animals showed little hemispheric asymmetry and no detectable c-Fos labeling at the injection site (M1/M2) or in more medial neocortical regions (Figure 2G).

A more moderate ipsilateral increase was observed in animals expressing the interneuron-targeted hDlx-hM3D(Gq) construct (Figures 2H–I).

Although based on a limited number of animals, these findings are consistent with successful chemogenetic modulation of cortical activity and support the interpretation of the imaging results.

To assess how chemogenetic stimulation of the right MC via DREADDs affects regional activation, we conducted Ph-fMRI during CNO infusion after a 15-minute baseline acquisition (Figure 3). The mean ipsilateral BOLD time courses during CNO uptake in MC of eDREADDs, iDREADDs, Shams and Saline controls (CTL) demonstrated significant differences across groups (Figure 3A). A two-way ANOVA with repeated measures was performed to compare the effects of Group (eDREADDs, iDREADDs, Shams, and CTL) and Time on the measured outcome. There was a significant Group × Time interaction (F_33,231_ = 10.65, *p* < 0.0001), indicating that the effect of Group differed across time points. Group alone had a significant effect (F_3,21_ = 14.32 *p* < 0.0001), and Time also significantly influenced the outcome (F_11,231_ = 30.53, *p* < 0.0001). The significant interaction suggests that comparisons between Groups at individual time points are more informative than overall main effects. Therefore, Bonferroni corrections were applied at each time point showing a highly significant difference (*p* < 0.001) between eDREADDs and iDREADDs time courses between 30 and 60 min during CNO passage. eDREADDs BOLD time courses were also significantly different from Shams and saline CTLs BOLD time courses (from the injection time (15 min) until the end of acquisition, *p* < 0.001). iDREADDs time courses were significantly different from Shams’s (*p* < 0.001 from 25 min on) and the saline CTLs (*p* < 0.001 at 44 min). Finally, Shams and Saline Controls presented no significant difference (*p* > 0.05).

**FIGURE 3:**
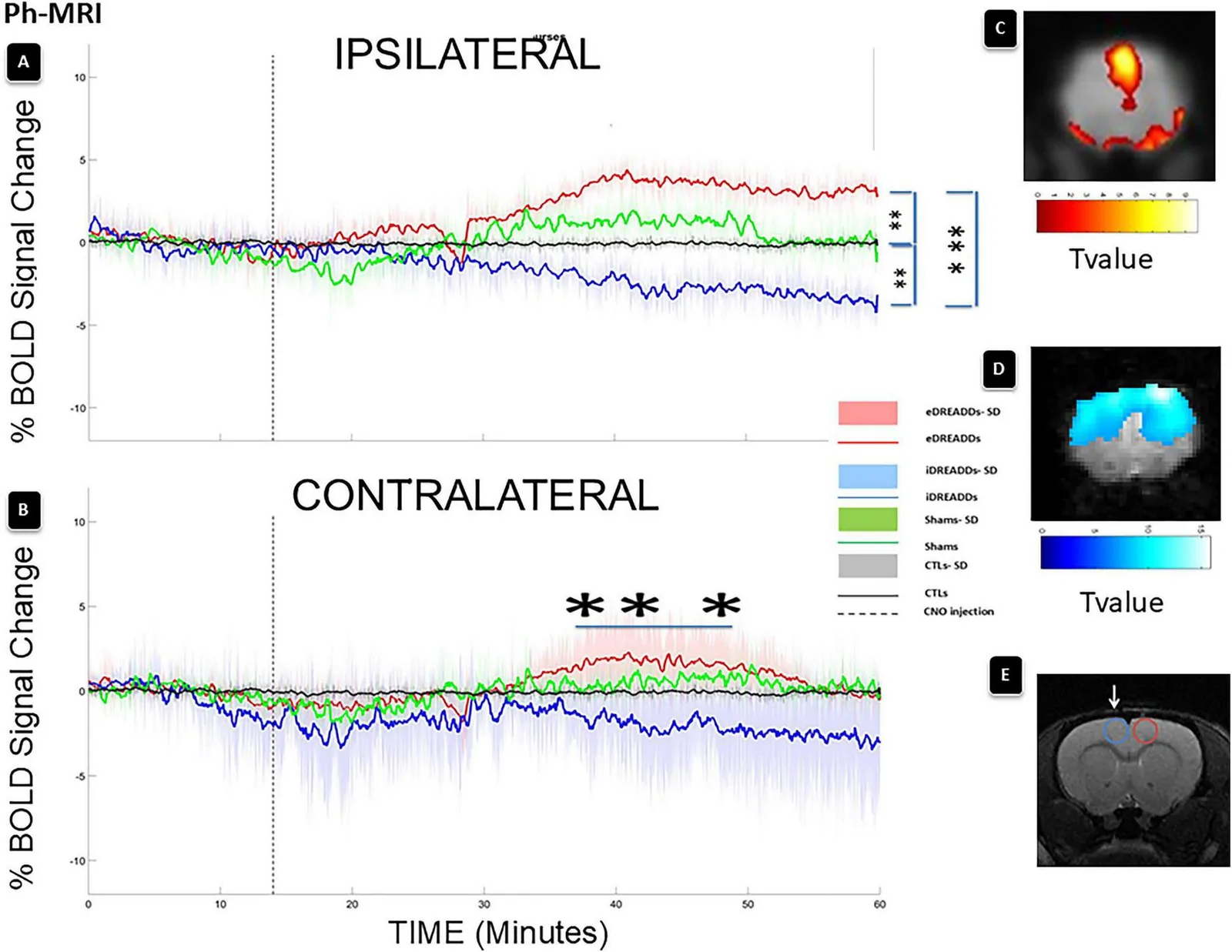
Chemogenetic modulation induces lateralized BOLD responses in the motor cortex. **(A)** Ipsilateral BOLD time courses following CNO administration (vertical dashed line). eDREADD animals show sustained positive BOLD responses (3.5 ± 1.5 % vs. 0.22 ± 0.03 %), whereas iDREADD animals exhibit progressive negative BOLD changes (−3.31 ± 1% vs. −0.042 ± 1%); 95 % CI [−3.75 −0.022]. Sham animals show a slight effect from 30 to 50 min post-CNO; 95 % CI [−0.65, 0.85]. Shaded areas: ±SD. Asterisks indicate significant group differences (*p < 0.05, **p < 0.01, ***p < 0.001). **(B)** Contralateral BOLD time courses showing attenuated but significant responses in DREADD groups. Horizontal bars denote significant time windows (**p < 0.01, ***p < 0.001). eDREADDs (1.13 ± 2.5 % vs. 0.02 ± 1%;95 % CI [−2 3.2]; iDREADDs (−2.7 ± 1.4 % vs. −0.17 ± 1.3 %; 95 % CI [−3.2 −0.04]; Shams: (0.73 ± 0.9 % vs. 0.05 ± 1.2 %) ; 95 % CI [−0.5 0.074]; CTL 95 % CI [−0.028 0,11]. **(C)** Statistical parametric maps (*t* values) illustrating positive BOLD activation in eDREADD animals. **(D)** Corresponding hemispheric comparison in iDREADD animals (trend toward lateralization during activation; *p* = 0.06). **(E)** Representative anatomical image showing voxel placement in the right motor cortex.

On the contralateral side (Figure 3B), a two-way repeated-measures ANOVA revealed a highly significant interaction between Time and Groups, indicating that the effect of Time varied across Groups (*F*_(33, 231)_ = 36.66, *p* < 0.0001). This interaction suggests that the temporal pattern of change differed meaningfully among groups. Nevertheless, Time also exhibited a very strong main effect (*F*_(11, 231)_ = 22.53, *p* < 0.0001), demonstrating a robust overall influence on the outcome. Groups also showed a significant main effect (*F*_(3, 21)_ = 13.71, *p* < 0.0001). Pos-hoc Bonferroni tests indicated significant differences between eDREADDs and iDREADDs contralateral BOLD time courses in the range 37–54 min (*p* < 0.001). No significant differences were found between BOLD time courses between eDREADDs and Shams or Controls (*p* > 0.05), while iDREADDS BOLD time courses showed significant differences against Shams and CTLs (*p* < 0.05 from 48 min). No significant difference was found between Shams and CTLs (*p* > 0.05 at all-time points). Consistent with the absence of DREADD expression, Sham animals did not show any significant difference (*p* > 0.05) between baseline and post-CNO periods, and their small drift is compatible with physiological variability under medetomidine–isoflurane sedation.

### 3.2 Ph- fMRI: excitatory DREADD manipulations produce opposite effects on contra–ipsi BOLD differences over time

Pharmacological fMRI revealed clear bidirectional modulation of cortical activity: in eDREADD animals, CNO induced strong focal positive BOLD activation in the right ipsilateral MC, extending to homotopic areas (IPSI: +3.5 ± 1.5% vs. CONTRA: +1.1 ± 2.5%; Figures 3C, 4A and Supplementary Figure 1). In contrast, interneuron activation DREADD (iDREADD) animals exhibited robust negative BOLD responses (IPSI: −3.3 ± 0.8% vs. CONTRA: −2.7 ± 0.8%; Figures 3D, 4B–F), while shams showed no significant changes (IPSI: +0.8 ± 1.5%; CONTRA: +0.7 ± 1.5%; Figure 4C). In Sham rats, the contra–ipsi difference at intercept was small (+0.26) and not statistically significant, indicating roughly equal BOLD responses in contralateral and ipsilateral sides. The difference remained essentially stable over time. eDREADD rats on the other end, showed a small but significant contra > ipsi difference at intercept (+0.30). Over time, the difference decreased slightly (slope = −0.053 per unit time). iDREADD rats exhibited a significant ipsi > contra difference at intercept (−0.92), which was significantly lower than eDREADD at baseline (*p* < 0.00001). Over time, the difference increased (slope = +0.029), indicating a gradual shift toward contra > ipsi. Between-group comparisons were performed showing that at baseline, iDREADD differed significantly from eDREADD, while Sham did not. The slopes over time were significantly different across all groups, reflecting distinct temporal dynamics: eDREADD decreased slightly, iDREADD increased, and Sham remained flat. Excitatory and interneuron activation DREADD manipulations produce opposite effects on contra–ipsi BOLD differences over time, whereas Sham rats show minimal lateralization.

**FIGURE 4:**
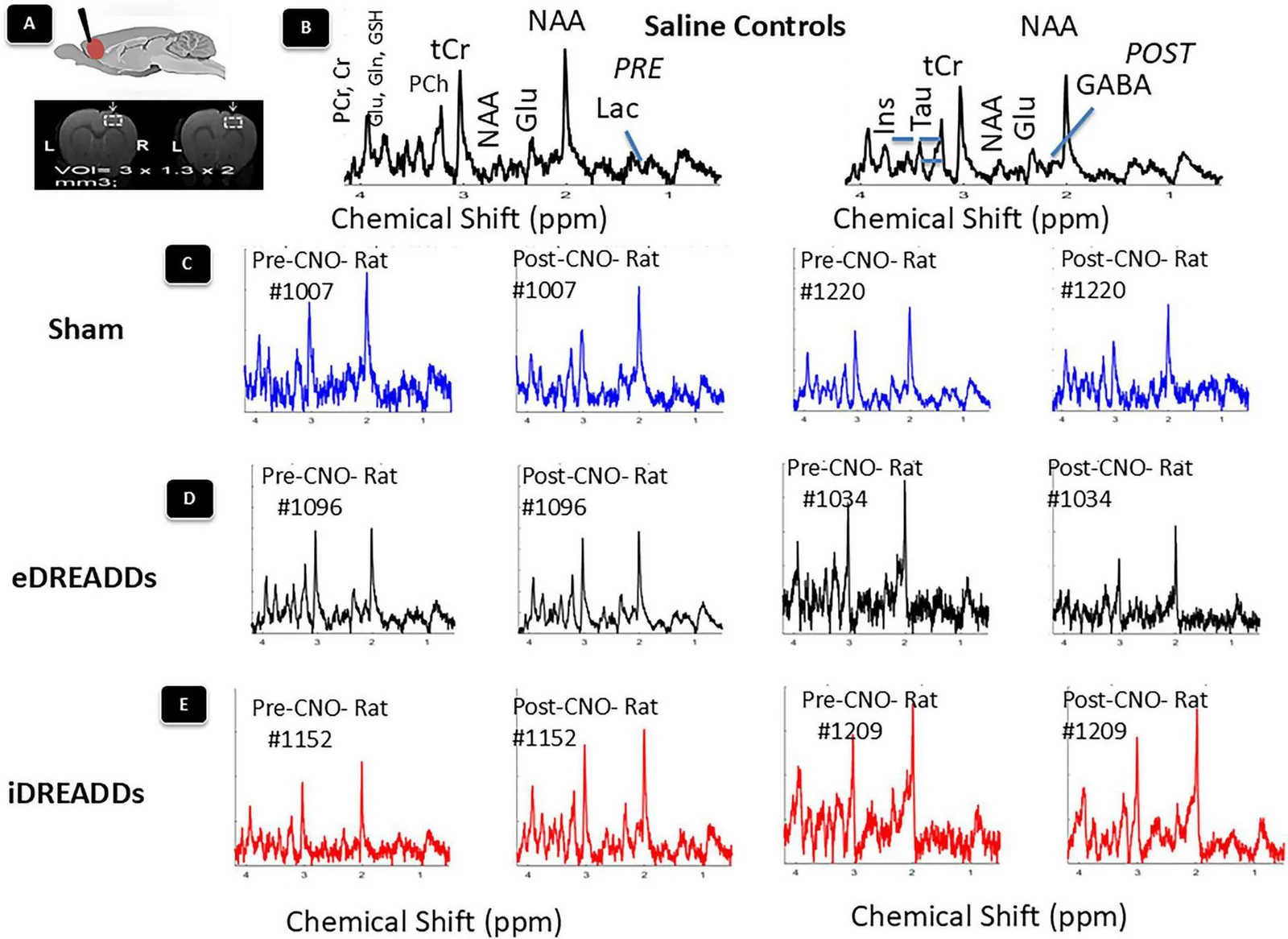
Functional metabolic responses measured with ^1^H fMRS during chemogenetic activation. **(A)** Schematic of the ^1^H fMRS voxel of interest (3 mm × 1.3 mm × 2 mm) positioned over the right motor cortex at the viral injection site, shown on coronal anatomical images. **(B)** Representative time-averaged spectra from saline controls before (Pre saline) and during (Post saline) acquisition. Major metabolite resonances (Glx, GABA, NAA, creatine compounds, choline-containing compounds) are labelled; #1xxx represent individual rat IDs. All spectra use the same vertical scale and were normalized to their respective water peak measured prior to the dynamic acquisition-**(C–E)** Representative Pre-CNO and Post-CNO spectra from Shams, eDREADD, and iDREADD animals acquired in the same cortical region. Two examples per group are shown. Spectral profiles are comparable across conditions. #1xxx represent individual rat IDs. LCModel fits examples can be found in Supplementary Figure 3. All groups: *n* = 6–8 rats. Spectra shown for illustration; quantitative analyses are presented in subsequent figures.

### 3.3 ^1^H-fMRS revealed no significant neurochemical changes upon CNO activation

Figure 5A illustrates the VOI position for ^1^H-fMRS acquisitions. Figure 5B illustrates representative examples of averaged and labeled ^1^H MR spectra acquired in this VOI during baseline and saline infusion in MC in a representative CTL animal pre and post-Saline. Then Shams (Figure 5C), eDREADDs (Figure 5D) and iDREADDs (Figure 5E) time-averaged ^1^H MR spectra are depicted for 2 representative animals per group pre and post-CNO to illustrate the variability across animals and groups. The tCr linewidth ranged between 10.6 ± 0.8 and 12.2 ± 0.2 Hz pre- and post-CNO, respectively. The variability across animals and groups was further illustrated in Figures 4D–F where the percent change relative to baseline is shown for Glx, GABA and tNAA across groups. Repeated ANOVAs, followed by Bonferroni corrections, demonstrated no significant differences across groups (*p* > 0.05). Further, the time-averaged metabolite concentrations were calculated during the baseline (0–15 min), during the transition period (16–30 min) and during the active period for CNO (>30 min) for tNAA, Glx, and GABA across groups as well as the SNR at baseline and post-injection (Saline and CNO). No significant concentration changes were found within groups. Only a trend toward decreased tNAA was observed in eDREADDs. Across groups, metabolite concentration and SNR variability were observed to be attributed to physiological changes during long scanning times (sedation, motion, CNO and saline injections) (Supplementary Figure 2). The mean CRLBs per group as a function of time can be found in the supplementary tables. Metabolite concentration time courses with 5-minute temporal resolution were obtained for Glx (Glu + Gln) (Figure 6A), GABA (Figure 6B), tNAA (Figure 6C), tCr (Figure 6D) and Asp (Figure 6E) during CNO uptake in MC for eDREADD and iDREADD, Sham and CTL rats and compared across groups. The time course for the Glx to GABA ratio (Figure 6F) was also calculated to explore the excitatory to inhibitory balance (E/I). Using a linear mixed model analysis (LMM), we tested for the overall difference in the average metabolite concentrations between the 4 groups across all time points, for the effect of time and for the interaction between group and time. Table 2 summarizes the outcomes of the LMM. Variability across animals was relatively high for some metabolites, particularly tNAA and tCr. Such variability is not unexpected in rodent fMRS studies, where measurements reflect bulk metabolite concentrations within relatively small brain volumes and are influenced by physiological state, anesthesia, and inter-animal differences in viral expression and chemogenetic activation.

**FIGURE 5:**
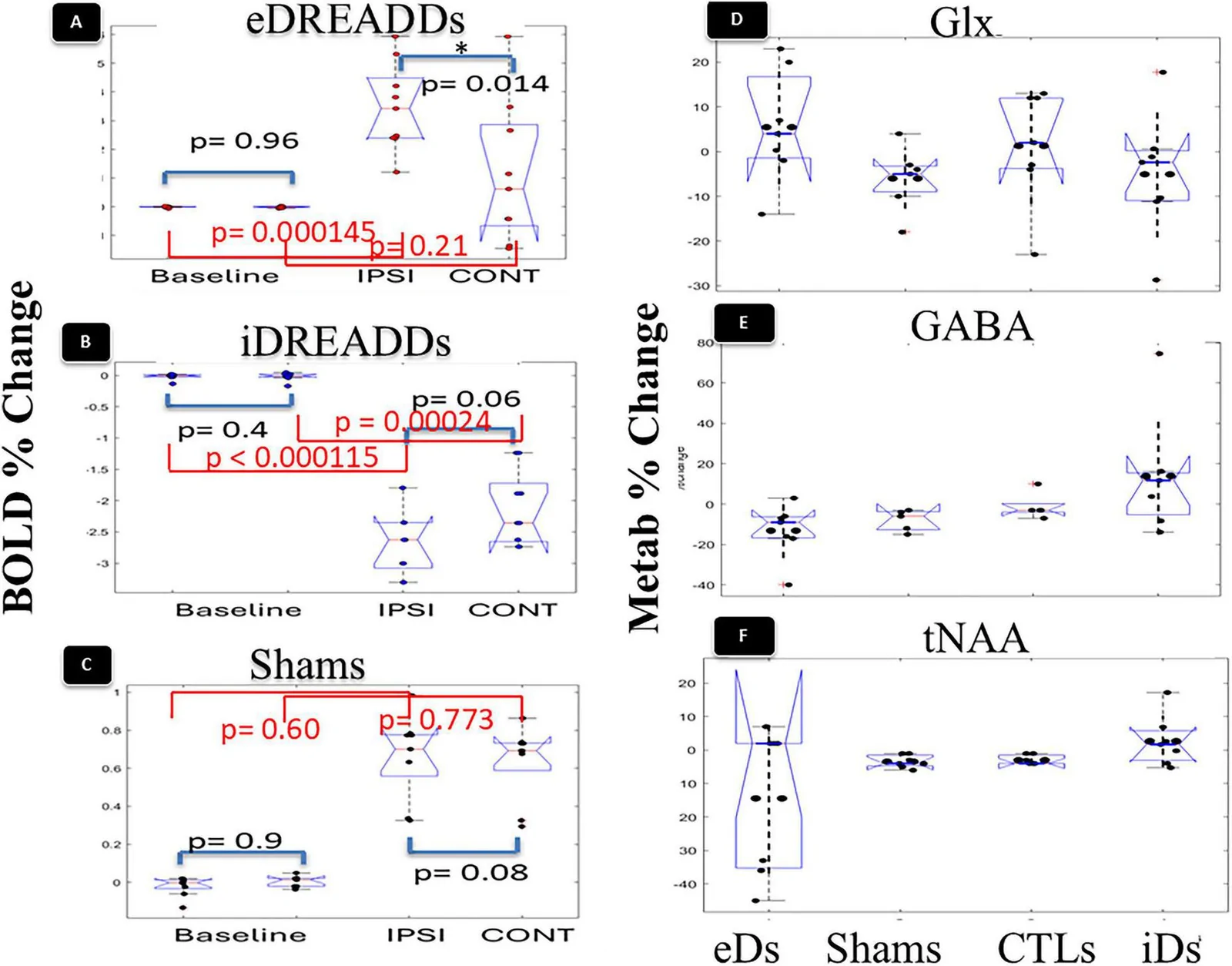
**(A)** Ipsilateral vs. contralateral BOLD signal in eDREADD animals at baseline and during activation (ipsilateral > contralateral during activation; *p = 0.014). Pre/Post comparison were also performed. *P*-values are indicated in red for IPSI/CONTRA (See Supplementary Figure 6). **(B)** Hemispheric comparison in iDREADD animals (trend toward lateralization during activation; *p* = 0.06). Pre/Post comparison were also performed. *P*-values are indicated in red for IPSI/CONTRA (See Supplementary Figure 6). **(C)** Sham animals show no hemispheric differences at baseline (*p* = 0.9) or activation (*p* = 0.08). Individual animals shown with group distributions. All groups: *n* = 6–8 rats. Pre/Post comparison were also performed. *P*-values are indicated in red for IPSI/CONTRA (See Supplementary Figure 6). **(D–F)** Time-averaged percent changes relative to baseline for Glx, GABA, and tNAA shown as boxplots with individual data points. Repeated-measures ANOVAs with Bonferroni correction revealed no significant group differences (*p* > 0.05).

**FIGURE 6:**
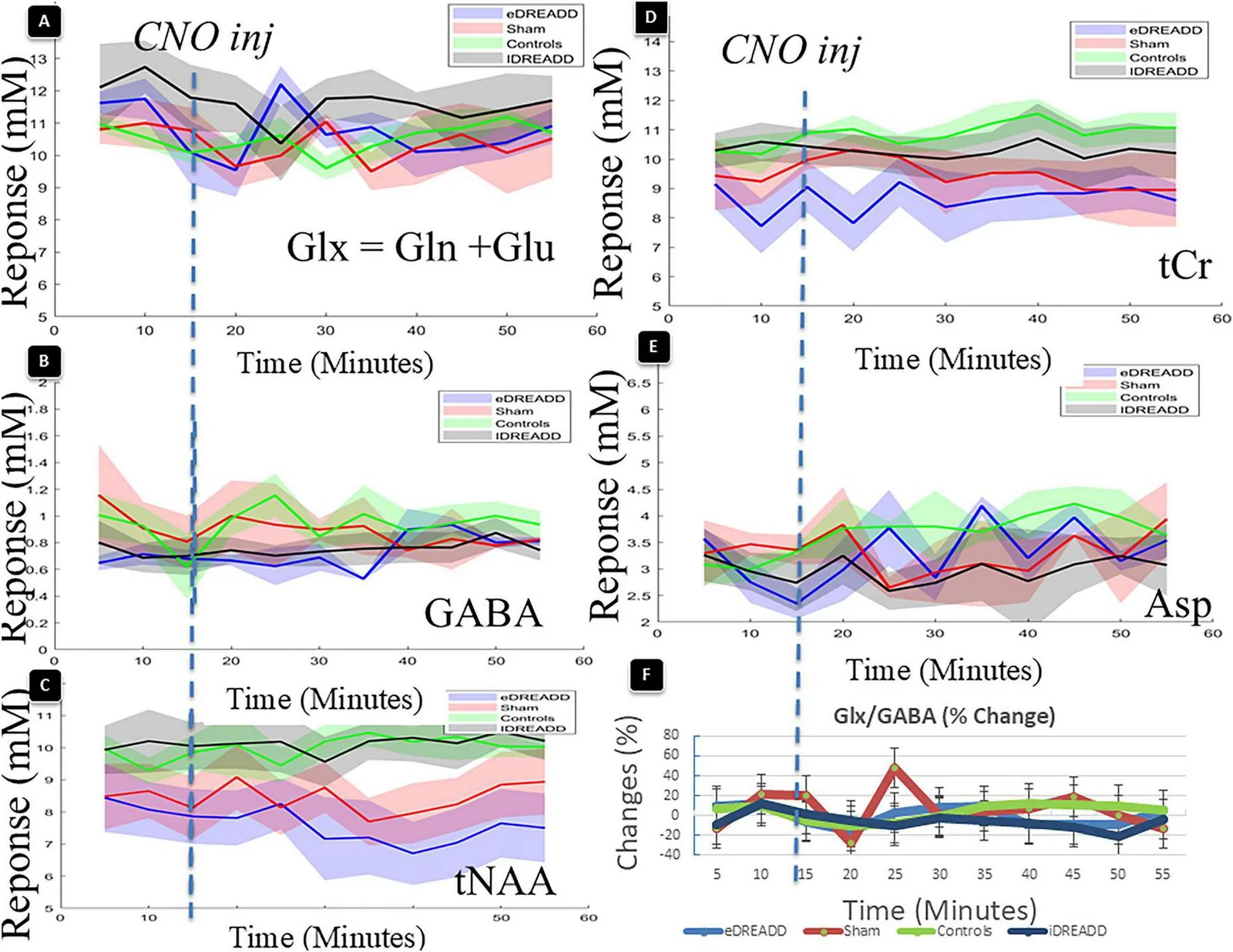
Time-resolved ^1^H fMRS during chemogenetic activation. **(A–E)** Group-averaged metabolite concentration time courses acquired from the right motor cortex before and after CNO injection (vertical dashed line). Traces show mean ± SD for panel **(A)** Glx, **(B)** GABA, **(C)** tNAA (NAA + NAAG), **(D)** total creatine (Cr + PCr), and **(E)** aspartate (Asp) in eDREADD, iDREADD, Sham, and saline control animals. Pre/Post Mean ± SD and 95% CI for each metabolite time course can be found in Supplementary Table 2. **(F)** Percent-change time courses for the Glx/GABA ratio (mean ± SD). Linear mixed-effects modeling (Table 2) indicated significant baseline group differences for Glx, GABA, and the Glx/GABA ratio, but no significant acute Group × Time effects following CNO for any metabolite. Minor time-dependent drifts for tNAA and total creatine were small and not specific to DREADD activation. All groups: *n* = 6–8 rats.

**TABLE 2:** LMM outcomes for raw metabolite concentration time courses: the chronic effect (the intercepts): the iDREADDs were in a fundamentally different metabolic state before any CNO was injected.

Metabolite	Model parameter	Comparison/ contrast	*P*-value (Holm)	Cohen’s d	Biological interpretation
Glx	Intercept	eDREADD vs. iDREADD	0.0047	−2.025	Baseline difference
Glx	Slope	Acute CNO effect	>0.05	<0.05	Functional uncoupling
	(15–59 min)				
GABA	Intercept	iDREADD vs. Sham	<0.01	−1.880	Baseline difference
GABA	Slope	Acute CNO effect	>0.05	<0.03	Functional uncoupling
Glx/GABA	Intercept	iDREADD vs. Sham	0.0088	2-342	Baseline E/I
Glx/GABA	Slope	Acute CNO effect	>0.05	<0.06	Stable E/I ratio
tCr	Intercept	iDREADDS vs.eDREADDs	0.142	−1.256	Baseline difference
tCr	Intercept	iDREADDs vs. Sham	0.626	0.652	Moderate baseline difference
tCr	Slope	Acute CNO effect	0.039	<0.05	Stability/minimal drift
tNAA	Intercept	eDREADDs vs. Saline CTL	*p* > 0.05	<0.05	All the groups
					had similar [tNAA] basline
tNAA	Slope	eDREADDs vs. Saline CTL	*p* = 0.048	<0.05	Signif. decreasing t[NAA] over time

With effect sizes |d|∼ 2.0, this isn’t just a minor statistical fluke; it is a major physiological shift in baseline Glx and GABA, pointing toward a chronic change likely induced by the viral expression itself (e.g., neuroinflammation or altered glial buffering). The acute effect (the slopes): the CNO injection, despite successfully triggering a BOLD response did not trigger changes on the bulk metabolite concentrations. The negligible effect sizes associated with acute CNO-induced changes (Cohen’s *d* < 0.1) indicate that bulk glutamatergic and GABAergic metabolite pools remained remarkably stable despite robust BOLD responses. These findings suggest that acute neurovascular responses can occur in the absence of detectable changes in bulk neurometabolic concentrations, consistent with an apparent degree of neurometabolic dissociation under the present experimental conditions.

For all the metabolites and the Glx to GABA ratio, the most important finding is the general lack of significant difference in the slopes (Group × time interaction term) after the CNO injection at 15 min (*post-hoc* slope analysis results). No significant differences were observed across groups for Glx, tNAA or tCr. eDREADD activation did not increase the rate of Glx production/consumption compared to saline-CTL and tCr was stable, suggesting CNO did not affect overall cellular energy balance or membrane integrity differently across groups. In addition, the CNO-driven effect did not shift the rate of E/I balance (Excitatory/Inhibitory) differently in DREADD groups although the rate of change of iDREADDs relative to eDREADDs and Shams were close to significance (*p* = 0.09 and *p* = 0.054, respectively). Only GABA concentration time courses showed significant differences between eDREADDs and Shams (*p* = 0.05) and iDREADDs and Shams (*p* = 0.009), respectively. The Shams were also initially different from CTL but not after Bonferroni correction.

### 3.4 The impact on basal metabolism

The significant differences at the baseline before CNO were the strongest findings of the present study. These results point toward long-term functional adaptation caused by the DREADD gene expression itself, before the CNO trigger: the basal Glx concentrations in eDREADDs were different from iDREADDs (*p* = 0.004), suggesting chronic differences in glutamatergic tone or basal energy state. The iDREADD group started with significantly lower GABA concentrations than the saline CTL group (*p* = 0.020). This is counter-intuitive and suggests that chronic expression of the iDREADD may lead to a compensatory downregulation of GABA. Hence, the iDREADD group had a significantly higher Glx to GABA ratio (or E/I balance) compared to all other groups. Although iDREADD is designed for inhibition, its baseline E/I balance is shifted toward excitation, likely due to the low basal GABA concentrations. The fact that tCr levels remained stable across all groups throughout the 60 min, with no significant slope differences, is also crucial: tCr is primarily an energy buffer. Its stability suggests that while neuronal energy demand is changing (based on BOLD), the overall supply of high-energy phosphates remains adequate and is not being acutely depleted or augmented differently in the DREADD groups.

### 3.5 Effect size analysis of Glx and GABA during baseline and CNO challenge

To complement inferential statistics, effect sizes (Cohen’s D) were computed for Glx and GABA concentration time courses during the baseline period and throughout the CNO challenge. For Glx, very large effect sizes were observed when comparing eDREADD and iDREADD groups, with Cohen’s D exceeding –2 across the entire time course, including during baseline and persisting throughout CNO administration. The magnitude of this effect remained stable over time and did not show further amplification following CNO injection (Supplementary Figure 4A). In contrast, comparisons between eDREADD and Sham animals yielded moderate-to-large effect sizes during baseline (Supplementary Figure 4B), which decreased during the CNO challenge, indicating a relative convergence of Glx levels following CNO administration. Across windows, effect sizes for eDREADD vs. Sham were substantially smaller than those observed for eDREADD vs. iDREADD. For GABA concentration timecourses, effect sizes were generally smaller than for Glx but followed a similar pattern. eDREADD vs. iDREADD comparisons produced consistently larger Cohen’s D values than comparisons involving Sham animals. While modest temporal fluctuations were observed during the CNO challenge, no systematic increase in effect size relative to baseline was detected. Together, these results indicate that the largest group separations in Glx and GABA were present prior to CNO administration, with acute CNO challenge producing limited additional separation at the level of bulk metabolite concentrations, despite robust group differences.

To assess whether the absence of detectable metabolic changes at 7T could be explained by sensitivity limitations rather than biological absence, we performed simulations of Glx and GABA functional time courses under identical chemogenetic perturbations at 7T and 14T. The simulations incorporated realistic pharmacokinetics, compartmental neurometabolic dynamics, and field-strength–dependent noise characteristics, while preserving the same underlying biological effect size across field strengths (See Supplementary data for Python codes, Supplementary Figure 5). These simulations should not be interpreted as a validation of the experimental findings. Rather, they were designed to explore whether biologically plausible neurometabolic changes that remain difficult to detect at 7 T could become more readily observable at higher magnetic field strengths. The results therefore provide a conceptual framework for interpreting the current data and generating hypotheses for future studies rather than definitive evidence regarding the mechanisms underlying the observed fMRS responses.

### 3.6 Late CNO/CLZ-driven changes (70 min post-CNO)

A two-way analysis of variance (ANOVA) was performed to assess the effect of acquisition site on neurometabolite concentrations approximately 70 min post-CNO administration. The analysis revealed an extremely significant interaction between the Hemisphere and Metabolite factors (*F*_(55, 408)_ = 17.95, *p* < 0.0001). This strong interaction demonstrates that the effect of the Hemisphere on metabolite concentration is not uniform; instead, the concentration of any given metabolite in a specific hemisphere is dependent on the type of metabolite being measured. Highly significant main effects were also observed for both Hemisphere (F _(5, 408)_ = 140.06, *p* < 0.0001) and Metabolite (*F*_(11, 408)_ = 35.13, *p* < 0.0001). Consequently, targeted post-hoc Bonferroni comparisons were performed to fully characterize the specific regional differences for each Metabolite-Hemisphere pair, which are presented below:

Within eDREADDs animals, several metabolites exhibited significant hemispheric asymmetries (Table 3). Specifically, GABA and Ins concentrations were significantly reduced in the contralateral hemisphere, whereas GPC+PCh was significantly increased contralaterally, indicating a shift affecting inhibitory tone and membrane turnover. In iDREADDs animals, hemispheric differences were more restricted (Table 3). A significant contralateral increase in GPC+PCh was observed, suggesting altered high-energy phosphate metabolism. In contrast, Sham animals displayed minimal hemispheric differences (Table 3), with only a limited number of metabolites reaching significance. Notably, Asp (+49.8%, *p* < 0.001) was increased contralaterally, confirming an overall symmetric metabolic profile in the absence of DREADDs expression. Between-group comparisons revealed marked differences in the extent and hemispheric distribution of metabolic alterations between eDREADDs and iDREADDs animals. Direct comparison between eDREADDs and iDREADDs animals revealed robust and widespread metabolic differences on both hemispheres (Table 4). These differences were markedly more extensive on the contralateral side. Across hemispheres, iDREADDs animals showed higher levels of high-energy phosphates and amino-acid–related metabolites relative to eDREADDs (Table 4). In eDREADDs rats, metabolic differences relative to Sham animals were limited on the ipsilateral side, with significant effects restricted to a small subset of metabolites (notably GABA, Ins, and GPC+PCh) (See Supplementary Table 1). In contrast, comparisons on the contralateral side revealed a larger number of significant alterations involving amino acids, energy-related metabolites, and phosphocholine compounds. In iDREADDs animals, comparisons with Sham animals revealed significant alterations across a broader range of metabolites on both hemispheres. These differences predominantly involved energy-related compounds such as glucose, and amino-acid–related metabolites (Asp, Glu+Gln, Tau, NAA+NAAG), indicating a more widespread metabolic divergence from Sham controls (See Supplementary Table 1).

**TABLE 3:** Hemispheric differences across groups (70 min post-CNO): comparison between contralateral and ipsilateral metabolite concentrations.

Metabolite	eDREADDs Δ (%)	SD	*p*	Sig	d	iDREADDs Δ (%)	SD	*p*	Sig	d	Sham Δ (%)	SD	*p*	Sig	d
Asp	−15.8	24.4	>0.05	ns	0.65	−1.78	13.6	0.05	ns	−0.18	+49. 8	8.9	0.001	***	7.9
GABA	−18	11.1	<0.05	*	0.85	+10.0	9.9	0.05	ns	1.43	+11. 5	1.6	0.05	ns	10.0
Glc	+7. 4	19.2	>0.05	ns	0.38	+6. 32	7.28	0.05	ns	1.23	+6. 4	3.0	0.05	ns	3.0
Ins	−29.5	11.4	<0.001	***	2.58	+4. 69	7.81	0.05	ns	0.85	+8. 3	2.8	0.05	ns	4.2
Tau	−17	10.6	<0.05	*	0.83	+4. 58	9.9	0.05	ns	0.65	−11.3	3.7	0.05	ns	−4.4
GPC+PCh	+20. 2	13.3	<0.01	**	0.87	+19. 8	5.62	0.01	**	1.79	−16	2.3	0.05	ns	−10.0
NAA+NAAG	−7.6	8.1	>0.05	ns	0.94	+0. 72	7.07	0.05	ns	0.14	+14. 4	2.1	0.05	ns	9.8
Glu+Gln	−4.1	17.0	>0.05	ns	0.24	+10. 84	4.24	0.05	ns	3.61	+3.0	1.0	0.05	ns	4.2
PCr +Cr	−4.8	6.0	>0.05	ns	0.84	−4.8	4.0	0.05	ns	−1.65	+9. 8	1.7	0.05	ns	8.2

*p*-values are Bonferroni-corrected (*n* = 12 comparisons per group). *p < 0.05; **p < 0.01; ***p < 0.001.

**TABLE 4:** Consolidated late-point metabolic comparisons (*n* = 44 comparisons).

Metabolite	Comparison	Dir	Δ (%)	SD	*P*-value	Sig	Cohen’s d
Asp	eD vs. iD (Ipsi)	↑	+20. 8	14.4	<0.01	**	1.44
	eD vs. iD (Contra)	↑	+34. 8	16.3	0.001	***	3.03
	eD vs. Sham (Ipsi)	↓	−4.0	17.0	ns	ns	−0.32
	eD vs. Saline (Ipsi)	↑	+14. 2	18.1	ns	ns	0.78
GABA	eD vs. iD (Ipsi)	↑	+15. 7	13.0	ns	ns	1.21
	eD vs. iD (Contra)	↑	+43. 8	19.3	0.001	***	3.30
	eD vs. Sham (Ipsi)	↑	+14. 4	4.8	ns	ns	3.00
	eD vs. Saline (Ipsi)	↑	+7. 3	5.1	ns	ns	1.43
Glc	eD vs. iD (Ipsi)	↑	+32. 9	9.2	<0.001	***	3.57
	eD vs. iD (Contra)	↑	+31. 8	17.1	0.001	***	2.73
	eD vs. Sham (Ipsi)	↑	+7.0	7.2	ns	ns	0.97
	eD vs. Saline (Ipsi)	↑	+14. 7	7.6	ns	ns	1.93
Ins	eD vs. iD (Ipsi)	↓	−16.4	12.2	<0.05	*	1.34
	eD vs. iD (Contra)	↑	+17. 8	10.3	0.05	*	2.5
	eD vs. Sham (Ipsi)	↓	−18.6	10.1	<0.05	*	−1.84
	eD vs. Saline (Ipsi)	≈	+1. 4	10.8	ns	ns	0.13
Tau	eD vs. iD (Ipsi)	↑	+13. 8	12.2	ns	ns	1.13
	eD vs. iD (Contra)	↑	+35. 4	12.2	0.001	***	4.15
	eD vs. Sham (Ipsi)	↓	−5.0	17.1	ns	ns	−0.29
	eD vs. Saline (Ipsi)	↓	−8.2	17.5	ns	ns	−0.47
GPC+PCh Cr+PCr	eD vs. iD (Ipsi)	↑	+31. 7	11.6	<0.001	***	2.73
	eD vs. iD (Contra)	↑	+31. 3	19.2	0.001	***	2.32
	eD vs. Sham (Ipsi)	↑	+57. 8	8.1	<0.001	***	7.13
	eD vs. Saline (Ipsi)	↑	+72. 4	8.5	<0.001	***	8.52
	eD vs. iD (Ipsi)	↑	+26. 5	5.0	<0.001	***	5.29
	eD vs. iD (Contra)	↑	+26. 5	7.1	0.001	***	5.50
	eD vs. Sham (Ipsi)	↑	+1. 1	4.0	ns	ns	0.28
	eD vs. Saline (Ipsi)	↑	+13.0	4.3	ns	ns	3.02
Glu+Gln	eD vs. iD (Ipsi)	↑	+9. 1	15	ns	ns	0.61
	eD vs. iD (Contra)	↑	+24. 1	5.0	0.001	***	6.88
	eD vs. Sham (Ipsi)	↓	−6.5	12.3	ns	ns	−0.53
	eD vs. Saline (Ipsi)	↓	−2.6	12.8	ns	ns	−0.20

*p*-values are Bonferroni-corrected for 44 comparisons. Direction (Dir) and percentage change (Δ %) refer to the first group relative to the second. eDREADDs (eD), iDREADDS (iD). *p < 0.05; **p < 0.01; ***p < 0.001.

In Saline control animals, hemispheric comparison performed approximately 70 min after injection revealed significant differences only for Glc, Asp (*p* < 0.05) and total choline (GPC+PCh, *p* < 0.05)) after Bonferroni correction, with no other metabolites affected. A similar restricted pattern was observed when Saline controls were compared with Sham animals on either hemisphere (See Supplementary Table 1). Thus, under control conditions, delayed static MRS reveals only minimal hemispheric metabolic asymmetry, indicating that the widespread alterations observed in DREADD-expressing animals are not attributable to injection-related or time-dependent effects. Relative to Saline controls, DREADD-expressing animals exhibited marked and asymmetric metabolic alterations at the delayed static MRS time point (∼70 min post-injection). In eDREADDs animals, the ipsilateral hemisphere differed from Saline animals (both ipsilateral and contralateral) primarily for total choline (GPC+PCh), which was significantly altered on both sides. In contrast, the contralateral hemisphere of eDREADDs animals showed broader deviations: compared with Saline-Ipsi, significant differences were observed for Asp, GABA, Ins, and total choline, whereas comparison with Saline-Contra revealed a significant effect only for GABA (See Supplementary Table 1). In iDREADDs animals, the ipsilateral hemisphere differed from Saline-Ipsi for Asp, Ins, and Tau, and from Saline-Contra for a wider panel of metabolites, including Asp, Glc, Tau, total NAA, and tCr. The contralateral hemisphere of iDREADDs animals exhibited the most extensive divergence, differing significantly from both Saline-Ipsi and Saline-Contra across the majority of measured metabolites. Thus, while Saline animals displayed only minimal hemispheric metabolic asymmetries, DREADD-expressing animals showed robust and hemisphere-dependent deviations, with iDREADDs animals exhibiting the broadest and most pronounced late metabolic alterations (See Supplementary Table 1). The mean CRLBs per group can be found in the Supplementary tables.

## 4 Discussion

The exact metabolic cost of DREADD-mediated circuit manipulation, particularly with respect to acute energy demand, neurotransmitter turnover, and neurovascular signalling, remains poorly understood. To address this gap, we combined longitudinal BOLD fMRI and ^1^H-fMRS in the motor cortex of sedated rats using a neuro-preserving imaging protocol (Just and Hoehn, 2025). Rather than attempting to isolate individual cellular contributions, we exploited the contrasting network-level consequences of chemogenetic recruitment of broad neuronal populations and preferential recruitment of inhibitory interneuron networks. This approach enabled us to investigate how distinct patterns of circuit engagement influence the relationship between neuronal activity, metabolism, and vascular responses.

Two major findings emerge from the present study. First, robust baseline neurometabolic differences were present between DREADD groups before ligand administration. Second, acute chemogenetic activation elicited large BOLD responses without detectable changes in bulk Glx or GABA concentrations during the first hour following CNO administration. Whereas these findings could straightaway be ascribed to sensitivity issues of ^1^H-fMRS at a preclinical field strength of 7T (Gruetter et al., 1998; Mlynárik et al., 2008), we also measured and detected significant metabolic interhemispheric changes at a later timepoint (70-min post-CNO) within the same animals.

Although these delayed metabolic changes may reflect, at least in part, the conversion of CNO to clozapine rather than direct DREADD activation (Gomez et al., 2017; Manvich et al., 2018) (discussed below), they nevertheless demonstrate that measurable metabolite concentration changes can be detected with the present MRS protocol. Their detectability therefore argues against a purely sensitivity-based explanation for the absence of significant acute metabolic changes during the period of robust BOLD activation.

Moreover, the comparison between 7 T and 14 T suggests that improved spectral resolution and signal-to-noise ratio at higher field strengths may enhance the detectability of subtle neurometabolic changes. However, the absence of significant acute fMRS responses at 7 T cannot be attributed solely to limited sensitivity. Alternative explanations include biological buffering of neurotransmitter pools, compartmental averaging effects, or apparent temporal dissociation between neuronal activity and measurable changes in bulk metabolite concentrations. Further supporting this, an earlier fMRSI study (Seuwen et al., 2015) also demonstrated a coupling mismatch between energy consumption and supply.

In the present study, chemogenetic recruitment of broad neuronal populations elicited pronounced positive BOLD responses, whereas preferential recruitment of inhibitory interneuron networks produced marked negative BOLD responses. Despite these opposing hemodynamic effects, neither manipulation induced proportional or consistent alterations in glutamatergic or GABAergic metabolite pools measured by ^1^H-fMRS during the CNO challenge. These findings suggest that substantial vascular responses can emerge in the absence of measurable changes in bulk neurotransmitter concentrations and suggest direct experimental evidence for the existence of a potential neurometabolic dissociation under conditions of externally driven circuit modulation. The contrasting responses observed between broad neuronal recruitment and interneuron-enriched recruitment further suggest that the magnitude and direction of the BOLD response depend strongly on the cellular composition of the engaged network (Gauthier and Fan, 2019; Uludağ, 2023). Conceptually, broad neuronal recruitment reflects the combined influence of excitatory and inhibitory neuronal populations, whereas interneuron-enriched recruitment preferentially emphasizes inhibitory network contributions (Dimidschstein et al., 2016; Hughes et al., 2025). Under this framework, the difference between these conditions may approximate the net excitatory component of the neurovascular response. However, because cortical circuits are highly recurrent and nonlinear, this interpretation should be considered a systems-level conceptual model rather than a direct measure of excitatory neuronal activity. Importantly, the absence of detectable neurotransmitter changes does not necessarily imply the absence of altered neurotransmission. Functional MRS measures changes in relatively large metabolite reservoirs (Just, 2021; Pasanta et al., 2023; Sonnay et al., 2018), whereas neuronal communication relies on rapid neurotransmitter release, uptake, recycling, and compartmental shifts (Zhang et al., 2026). Proton fMRS also measures net changes in metabolite concentrations rather than neurotransmitter flux. Consequently, substantial alterations in glutamatergic and GABAergic signalling may occur without detectable changes in total metabolite pools. For example, increased neurotransmitter release may be accompanied by proportionate increases in reuptake and recycling through the glutamate-glutamine cycle, resulting in altered turnover rates but stable steady-state concentrations (Andersen, 2025; Sonnay et al., 2018). Similarly, ^1^H-MRS cannot readily distinguish among vesicular, cytosolic, extracellular, neuronal, and astrocytic neurotransmitter pools. Because glutamate is present at millimolar concentrations and serves both neurotransmitter and metabolic functions, the measured signal largely reflects the aggregate contribution of multiple intracellular compartments rather than synaptically active transmitter pools (Rae, 2014; Sonnay et al., 2018). Additional complexity arises from the cellular compartmentalization of glutamate and GABA metabolism. A substantial fraction of cortical glutamate is located within neurons and astrocytes, where it participates in intermediary metabolism, protein synthesis, and neurotransmitter recycling. Consequently, localized synaptic changes may represent only a small fraction of the total metabolite pool visible to MRS. Changes in neurotransmission may therefore be buffered by large intracellular reservoirs and remain undetectable at the level of bulk concentration measurements (Andersen, 2025; Sonnay et al., 2018).

Temporal resolution also constitutes an important limitation. Neurotransmitter release, uptake, and recycling occur on timescales of milliseconds to seconds, whereas fMRS generally requires averaging over several minutes to achieve sufficient signal-to-noise ratio (Just, 2021; Pasanta et al., 2023). Rapid transient changes may therefore be averaged out, particularly when measurements are acquired from relatively large voxels encompassing heterogeneous neuronal populations. Under such circumstances, significant alterations in neurotransmitter dynamics may occur without producing measurable changes in the time-averaged metabolite concentrations estimated by ^1^H-fMRS.

Consequently, substantial changes in neurotransmitter flux and synaptic activity may occur without producing measurable alterations in steady-state glutamate or GABA concentrations. The present findings therefore suggest that the observed BOLD responses are not simply determined by bulk neurotransmitter pool size but instead reflect broader neurovascular processes. One potential explanation for this apparent dissociation is the differential engagement of the neurovascular unit. Astrocytes, pericytes, endothelial cells, and vascular smooth muscle cells collectively contribute to the translation of neuronal activity into local hemodynamic responses (Brookshier and Lyden, 2025; Hall et al., 2014; McConnell and Mishra, 2022). Astrocytes are of particular interest because they respond to both glutamatergic and GABAergic signaling and can regulate cerebral blood flow through calcium-dependent release of vasoactive mediators (Andersen, 2025). Under this framework, distinct patterns of neuronal recruitment may generate markedly different vascular responses despite producing minimal changes in measurable neurotransmitter concentrations. The positive BOLD responses associated with broad neuronal recruitment and the negative responses observed following preferential inhibitory network recruitment may therefore reflect differential engagement of neurovascular signaling pathways rather than differences in steady-state neurotransmitter levels alone. This interpretation aligns with a growing body of literature challenging the assumption of a tight and linear relationship between neuronal activity, metabolism, and hemodynamics. Pereira et al. (2026) recently demonstrated a dissociation between metabolic and hemodynamic responses during chemogenetic manipulation of astrocytes in the mouse visual cortex. Previous rodent studies have similarly shown that BOLD, cerebral blood flow, and cerebral blood volume responses do not always scale proportionally with neuronal or metabolic activity. For example, hippocampal seizures can generate large increases in neuronal firing and perfusion while producing paradoxical negative BOLD responses (Schridde et al., 2008), suggesting that oxygen consumption may outpace vascular delivery. Likewise, perforant-path stimulation induces prolonged hemodynamic depressions despite persistent after-discharges (Arboit et al., 2024), indicating the existence of multiple and potentially independent neurovascular coupling mechanisms. Pharmacological studies have further demonstrated that vascular responses depend strongly on the receptor systems engaged, with NMDA- and AMPA-mediated signalling producing distinct hemodynamic consequences despite ongoing neuronal excitation (Desmond et al., 1991). Together, these findings indicate that coupling between vascular signals and glutamate or GABA metabolism is conditional, context-dependent, and influenced by multiple cellular pathways. In humans, uncoupling is implicated in aging and neurodegenerative disorders such as Alzheimer’s disease, where pericyte loss, astrocytic dysfunction, and oxidative stress impair vascular dilation and metabolic efficiency (Graves and Baker, 2020). This has implications for the interpretation of combined fMRI–fMRS studies, particularly those aiming to infer neurotransmitter dynamics from BOLD signals (Just and Faber, 2019; Just and Sonnay, 2017; Just et al., 2013; Ligneul et al., 2021; Seuwen et al., 2015). The present results argue that caution is warranted when equating hemodynamic changes with neurotransmitter metabolism, especially in experimental paradigms involving neuromodulatory or chemogenetic interventions. The convergence of evidence across different cell types (neurons and astrocytes), cortical regions (motor and visual cortex), species, and experimental paradigms suggest that neurometabolic dissociation may represent a fundamental biological feature of externally driven circuit perturbations rather than a methodological artifact. Because DREADD-mediated modulation produces sustained cellular recruitment that differs from endogenous activity patterns, vascular responses may be strongly influenced by downstream vasoactive signalling pathways without necessarily requiring large-scale shifts in total neurotransmitter reservoirs. Collectively, our results support the view that hemodynamic signals emerge from interactions among multiple components of the neurovascular unit and that BOLD and fMRS measurements capture complementary but distinct aspects of circuit function.

### 4.1 Baseline metabolic differences

Beyond the acute chemogenetic effects, perhaps the most unexpected finding of the present study was the presence of substantial neurometabolic differences at baseline between eDREADD- and iDREADD-expressing animals. Indeed, the largest effect sizes observed in the entire dataset occurred prior to CNO administration, suggesting that long-term adaptations associated with DREADD expression may represent a more prominent neurometabolic signature than the acute effects of receptor activation. While the primary objective of this study was to characterize the acute hemodynamic and metabolic consequences of chemogenetic stimulation, these baseline differences may constitute an equally important biological observation and warrant careful consideration.

An important observation emerging from the present dataset is that substantial neurometabolic differences were already present at baseline between eDREADDs- and iDREADDs-expressing animals, particularly for Glx and related metabolites, with very large effect sizes (Cohen’s *d* > 2). The large effect sizes observed at baseline (Cohen’s *d* > 2) reflect substantial separation between chronically expressing DREADD groups relative to within-group variability and are therefore consistent with a marked neurometabolic divergence present prior to ligand administration. These differences precede ligand administration and therefore cannot be attributed to acute chemogenetic activation, CNO pharmacodynamics, or clozapine back-metabolism (Gomez et al., 2017; Manvich et al., 2018). Instead, they suggest that chronic expression of the constructs may contribute to distinct neurometabolic states. Such baseline divergence is biologically plausible. Sustained expression of excitatory versus inhibitory DREADDs can alter intrinsic excitability, synaptic balance, and network tone over weeks, even in the absence of ligand (Gomez et al., 2017; Manvich et al., 2018; Roth, 2016; Urban and Roth, 2015). Chronic perturbations of neuronal excitability are known to trigger homeostatic plasticity, synaptic scaling, and compensatory changes in excitation–inhibition balance (Davis, 2006; Turrigiano, 2012; Urban and Roth, 2015), processes that are metabolically costly and can reshape bulk pools of amino acids and high-energy phosphates. In parallel, long-term viral transduction itself can modify cellular physiology, glial engagement, and local metabolic demand (Keck et al., 2013). In this context, ^1^H-MRS captures a trait-like metabolic phenotype associated with circuit identity rather than a state-like response to acute activation (Rae, 2014). This interpretation reframes the largely negative dynamic ^1^H-fMRS findings: the dominant chemogenetic effect detectable at 7 T is not a transient modulation during the CNO challenge, but a stable reconfiguration of neurometabolic baseline. Acute BOLD responses then unfold on top of these pre-existing metabolic landscapes. Importantly, the magnitude of these baseline differences exceeded that of the acute CNO-induced metabolic effects. This suggests that the dominant metabolic consequence of the chemogenetic intervention may arise from long-term adaptations occurring during the weeks of construct expression, rather than from the transient receptor activation measured during the fMRS experiment. Consequently, the absence of significant acute changes in bulk metabolite concentrations should not necessarily be interpreted as evidence for an absence of metabolic consequences, but rather as indicating that the most robust detectable effects may already be embedded within a chronically altered metabolic state.

The apparent mismatch between large baseline differences and minimal dynamic changes suggests that chemogenetic constructs primarily bias long-term metabolic organization, while short-term vascular and electrophysiological responses are mediated through mechanisms that do not require proportional shifts in bulk neurotransmitter pools (Emir et al., 2011; Hall et al., 2014; Stiernman et al., 2023). Importantly, the use of a broad hSyn promoter for excitatory DREADDs may have further contributed to this effect by engaging heterogeneous neuronal populations and inducing diffuse homeostatic adaptations (Dimidschstein et al., 2016; Epp et al., 2026). Future work using cell-type–specific promoters and longitudinal pre- and post-transduction MRS will be necessary to disentangle viral-expression effects from ligand-driven neuromodulation.

A further limitation concerns the choice of the sham construct. Rather than using a fluorophore-only vector, we employed AAV5-CaMKIIa-hChR2(H134R)-EYFP as a viral expression control. This design was chosen to control for stereotaxic surgery, viral transduction, long-term transgene expression, and expression of an exogenous membrane protein in the absence of optical stimulation. Nevertheless, ChR2 expression cannot be considered fully biologically inert. Chronic membrane expression of channelrhodopsins has the potential to alter neuronal physiology, excitability, or network homeostasis independently of optical activation (Miyashita et al., 2013). Therefore, we cannot completely exclude the possibility that some baseline neurochemical or physiological differences between groups were influenced by long-term transgene expression. Although sham animals did not exhibit detectable c-Fos activation, robust BOLD responses, or the widespread metabolic alterations observed in DREADD-expressing animals, future studies incorporating fluorophore-only controls (e.g., mCherry or EYFP alone) would help further disentangle the effects of DREADD signalling from those associated with chronic expression of exogenous membrane proteins.

In contrast to the acute ^1^H-fMRS results, interhemispheric metabolic differences were observed approximately 70 min after CNO administration, when measurements were performed using static ^1^H-MRS rather than ^1^H-fMRS. Importantly, this time window overlaps with the emergence of measurable clozapine concentrations resulting from CNO back-conversion (Gomez et al., 2017; Manvich et al., 2018), complicating attribution of these effects to DREADD activation alone.

These late effects were not temporally aligned with the BOLD responses and therefore likely reflect secondary or delayed metabolic processes rather than direct consequences of acute circuit activation. Crucially, the delayed emergence of hemispheric asymmetries suggests that these metabolic changes are not causally linked to the BOLD dynamics observed earlier, reinforcing the interpretation of an apparent neurometabolic dissociation at acute timescales. A critical consideration in interpreting the delayed metabolic effects is the known back-metabolism of CNO to clozapine in rodents (Gomez et al., 2017; Manvich et al., 2018). Clozapine exhibits high affinity for multiple endogenous receptors and can exert widespread neuromodulatory and metabolic effects independent of DREADDs activation (Kügler et al., 2003; Morrison et al., 2025; Sternson and Roth, 2014). The timing of the observed interhemispheric differences is compatible with a scenario in which clozapine accumulation contributes to delayed metabolic alterations. While the present study was not designed to directly dissociate CNO- from clozapine-mediated effects, the apparent temporal mismatch between BOLD, ^1^H-fMRS, and static MRS findings strongly suggests that late metabolic changes should be interpreted with caution. For this reason, these results are best viewed as hypothesis-generating rather than definitive evidence of “chemogenetically” driven metabolic reorganization. The observation that delayed metabolic changes were often more pronounced contralaterally further supports the idea that these effects arise from distributed network processes or systemic influences, rather than localized neuronal activation. Importantly, Sham animals did not exhibit comparable hemispheric asymmetries, indicating that these effects are not attributable to measurement bias or baseline lateralization. However, given their delayed onset and the pharmacokinetic considerations discussed above, these interhemispheric differences likely reflect indirect consequences of chemogenetic manipulation and/or clozapine exposure, rather than primary neurometabolic correlates of BOLD activation.

As multimodal neuroscience continues to develop, addressing the underlying biophysical relationships between imaging modalities may prove just as important as advancing the measurement technologies themselves. To move beyond purely observational neuroimaging, the field must leverage causal perturbation methods, such as chemogenetics, optogenetics, transcranial magnetic stimulation (TMS), transcranial focused ultrasound (TFUS), and genetically encoded biosensors (Aggarwal et al., 2026) specifically to address how these independent axes of neurovascular and neurometabolic coupling operate.

## 5 Conclusion

In this study, chemogenetic excitation elicited large, opposing BOLD responses, yet these rapid hemodynamic changes were not accompanied by contemporaneous alterations in glutamate or GABA concentrations measured with ^1^H-fMRS at 7T. Rather than undermining the utility of fMRS, these results highlight the complementary information provided by hemodynamic and neurochemical imaging modalities. The absence of detectable acute metabolite changes despite robust BOLD responses suggests that neurovascular and neurometabolic readouts may not always evolve in parallel, cautioning against directly inferring acute neurotransmitter turnover from BOLD dynamics alone. Our work highlights that neurovascular and neurometabolic readouts can be experimentally dissociated. It provides a critical roadmap that motivates future multimodal studies to move beyond static pool sizes at standard field strengths, leveraging next-generation stable ligands, ultra-high fields (>14T), and dynamic flux kinetics to truly map out the independent pathways governing neurovascular and neurometabolic coupling.

## Data Availability

The raw data supporting the conclusions of this article will be made available by the authors upon reasonable request.

## Footnotes

1
http://nifti.nimh.nih.gov

2
https://www.s-provencher.com/lcmodel.shtml

3
https://fsl.fmrib.ox.ac.uk/fsl/fslwiki/

4
https://www.fil.ion.ucl.ac.uk/spm/

5
http://www.s-provencher.com/pages/lcmodel.html
