# Supplementary material for: Apparent dissociation between hemodynamic and neurochemical responses during chemogenetic modulation of cortical circuits in rats
Source: Front Neurosci. 2026 Oct 8;20:1916498. doi: 10.3389/fnins.2026.1916498 (PMC13686830; doi:10.3389/fnins.2026.1916498)
Supplement: Supplementary file 2 [file Supplementary_file_2.docx]

% Matlab Code for Spectra

clear; clc; close all;

%% 1. Parameters

n = 200; npts = 2048; BW = 4000; B0 = 300.344;

target_ppm = 4.68; lb = 1; npts_zf = 16384;

shift_pts_digital = 68;

avg_window = 9;

t = (0:npts-1)'/BW;

hz_per_ppm = B0;

%% 2. Load Data

myFolder = uigetdir();

if myFolder == 0, return; end

fname_raw = fullfile(myFolder, 'fid');

fid_raw = fopen(fname_raw, 'r');

[DataIn, ~] = fread(fid_raw, [2*npts, n], 'int32');

fclose(fid_raw);

fids = complex(DataIn(1:2:end, :), DataIn(2:2:end, :));

%% 3. Alignment & Phasing

f_axis = linspace(-BW/2, BW/2, npts)';

phc1_ramp = exp(1i * 2 * pi * f_axis * (shift_pts_digital / BW));

ppm_scale = linspace(-BW/2, BW/2, npts) / B0 + target_ppm;

fids_corr = zeros(npts, n);

for k = 1:n

spec = fftshift(fft(fids(:,k))) .* phc1_ramp;

fid = ifft(ifftshift(spec));

s_abs = abs(fftshift(fft(fid .* exp(-15*pi*t))));

[~, max_i] = max(s_abs);

df = (target_ppm - ppm_scale(max_i)) * B0;

fid = fid .* exp(1i * (2 * pi * df * t));

temp_spec = fftshift(fft(fid .* exp(-lb*pi*t)));

[~, naa_idx] = min(abs(ppm_scale - 2.01));

anchor_phase = angle(temp_spec(naa_idx));

fids_corr(:,k) = fid * exp(-1i * anchor_phase);

end

%% 4. SNR & Block Analysis

% Define Block 1 (Before) and Block 2 (After)

fid_blocks = {1:60, 120:200};

snr_values = zeros(length(fid_blocks), 1);

ppm_zf = linspace(-BW/2, BW/2, npts_zf) / B0 + target_ppm;

figure('Color', 'w', 'Position', [100, 100, 1000, 500]);

for b = 1:length(fid_blocks)

% 1. Average the FIDs in the block

avg_fid = mean(fids_corr(:, fid_blocks{b}), 2) .* exp(-lb*pi*t);

spec_raw = real(fftshift(fft(avg_fid, npts_zf)));

% 2. Signal: NAA Peak Amplitude (2.01 ppm)

sig_mask = ppm_zf > 1.9 & ppm_zf < 2.1;

naa_amp = max(spec_raw(sig_mask));

% 3. Noise: SD of baseline (5.0 to 8.0 ppm)

noise_mask = ppm_zf > 5.0 & ppm_zf < 8.0;

noise_floor = spec_raw(noise_mask);

noise_sd = std(detrend(noise_floor)); % Detrend to remove baseline drift

% 4. Absolute SNR Calculation

snr_values(b) = naa_amp / noise_sd;

% Plotting for Visual Verification

subplot(1, 2, b)

plot(ppm_zf, spec_raw, 'k'); hold on;

fill([5 8 8 5], [min(ylim) min(ylim) max(ylim) max(ylim)], 'y', 'FaceAlpha', 0.1, 'EdgeColor', 'none');

set(gca, 'XDir', 'reverse', 'Box', 'off'); xlim([0.5 8.5]);

title(sprintf('Block %d (SNR = %.2f)', b, snr_values(b)));

xlabel('ppm'); ylabel('Intensity');

if b == 1, text(6.5, max(ylim)*0.8, 'Noise Region', 'Color', 'k', 'FontWeight', 'bold'); end

end

##Simulations

import numpy as np

from scipy.integrate import odeint

import matplotlib.pyplot as plt

t = np.linspace(0, 120, 121)

CNO_onset, CNO_duration = 15, 60

tau = 5

\# Baselines & Noise

Glx0_7, GABA0_7 = 10.0, 0.8

Glu0_14, GABA0_14 = 12.0, 1.5

n7, n14 = 1.0, 0.15

\# Excitatory Effect

E_glx, E_gaba = 0.2, -0.05

def CNO(t): return 1.0 * (t >= CNO_onset) * (t <= CNO_onset + CNO_duration)

def solve_mrs(base, e_max, noise_lvl):

clean = odeint(lambda y, t: (base * (1 + e_max * CNO(t)) - y) / tau, base, t).flatten()

return clean + np.random.normal(0, noise_lvl, len(t))

def get_cohens_d(data, window=7):

baseline = data[0:15]

mu, sigma = np.mean(baseline), np.std(baseline, ddof=1) + 1e-6

d_raw = (data - mu) / sigma

return np.convolve(d_raw, np.ones(window)/window, mode='same')

fig1, ax = plt.subplots(2, 2, figsize=(14, 10))

def format_plot(axis, title, ylabel):

axis.axvspan(CNO_onset, 75, color='gray', alpha=0.15, label='CNO Active')

axis.set_title(title)

axis.set_ylabel(ylabel)

axis.set_xlabel("Time (min)")

axis.legend(loc='upper right')

\# Generate Data

glx_7, gaba_7 = solve_mrs(Glx0_7, E_glx, n7), solve_mrs(GABA0_7, E_gaba, n7)

glu_14, gaba_14 = solve_mrs(Glu0_14, E_glx, n14), solve_mrs(GABA0_14, E_gaba, n14)

\# Plotting

ax[0,0].plot(t, glx_7, label='Glx', color='royalblue')

ax[0,0].plot(t, gaba_7, label='GABA', color='crimson')

format_plot(ax[0,0], "7T Metabolite Concentrations", "Conc (a.u.)")

ax[0,1].plot(t, get_cohens_d(glx_7), label="d-Glx", color='royalblue')

ax[0,1].plot(t, get_cohens_d(gaba_7), label="d-GABA", color='crimson')

format_plot(ax[0,1], "7T Cohen's D (Smoothed)", "Effect Size (d)")

ax[1,0].plot(t, glu_14, label='Glu', color='teal')

ax[1,0].plot(t, gaba_14, label='GABA', color='darkred')

format_plot(ax[1,0], "14T Metabolite Concentrations", "Conc (a.u.)")

ax[1,1].plot(t, get_cohens_d(glu_14), label="d-Glu", color='teal')

ax[1,1].plot(t, get_cohens_d(gaba_14), label="d-GABA", color='darkred')

format_plot(ax[1,1], "14T Cohen's D (Smoothed)", "Effect Size (d)")

plt.tight_layout()

snr_levels = np.linspace(5, 60, 15)

def snr_sweep(base, e_max):

d_scores = []

\# Clean curve for reference

clean = odeint(lambda y, t: (base * (1 + e_max * CNO(t)) - y) / tau, base, t).flatten()

for s in snr_levels:

noise_sigma = base / s

trials = []

for _ in range(20):

noisy = clean + np.random.normal(0, noise_sigma, len(t))

d = (np.mean(noisy[40:110]) - np.mean(noisy[0:15])) / (np.std(noisy[0:15]) + 1e-6)

trials.append(abs(d))

d_scores.append(np.mean(trials))

return d_scores

plt.figure(figsize=(12, 5))

plt.subplot(1,2,1)

plt.plot(snr_levels, snr_sweep(Glx0_7, E_glx), 'o-', label='Glx (7T)')

plt.plot(snr_levels, snr_sweep(GABA0_7, E_gaba), 's-', label='GABA (7T)')

plt.title("7T: SNR vs. Detectability")

plt.xlabel("SNR (Mean/Noise SD)")

plt.ylabel("Measured Cohen's d")

plt.legend()

plt.grid(True, alpha=0.3)

plt.subplot(1,2,2)

plt.plot(snr_levels, snr_sweep(Glu0_14, E_glx), 'o-', label='Glu (14T)', color='teal')

plt.plot(snr_levels, snr_sweep(GABA0_14, E_gaba), 's-', label='GABA (14T)', color='darkred')

plt.title("14T: SNR vs. Detectability")

plt.xlabel("SNR (Mean/Noise SD)")

plt.ylabel("Measured Cohen's d")

plt.legend()

plt.grid(True, alpha=0.3)

plt.tight_layout()

plt.show()

Python Code for Simulations at 7 and 14T

import numpy as np

import matplotlib.pyplot as plt

from scipy.integrate import odeint

\# ==========================================

\# 1. PARAMETERS

\# ==========================================

t = np.linspace(0, 120, 241) # 120 minutes, 0.5 min steps

CNO_onset = 15

CNO_offset = 100

tau = 5.0 # Time constant (minutes)

\# 7T Baselines & Effects

base_7T = {'Glx': 10.0, 'GABA': 0.8}

E_max_7T = {'Glx': -0.10, 'GABA': 0.15} # Glx decreases, GABA increases

\# 14T Baselines & Effects

base_14T = {'Glu': 12.0, 'Gln': 2.0, 'GABA': 1.5}

E_max_14T = {'Glu': -0.12, 'Gln': -0.03, 'GABA': 0.10}

\# Noise levels (Standard Deviation)

noise_7T = 0.4

noise_14T = 0.1 # 14T typically has higher SNR/lower noise

\# ==========================================

\# 2. FUNCTIONS

\# ==========================================

def indicator_CNO(t):

"""Binary switch for CNO activation."""

return 1.0 if CNO_onset <= t <= CNO_offset else 0.0

def metabolite_ode(C, t, C0, E_max):

"""

Implements: dC/dt = (C0 * (1 + E_max * I(t)) - C) / tau

"""

target = C0 * (1 + E_max * indicator_CNO(t))

dC_dt = (target - C) / tau

return dC_dt

\# ==========================================

\# 3. SOLVING

\# ==========================================

def simulate_system(baselines, effects, noise_lvl):

results = {}

for name in baselines.keys():

\# Solve ODE

clean_signal = odeint(metabolite_ode, baselines[name], t,

args=(baselines[name], effects[name])).flatten()

\# Add Gaussian Noise

results[name] = clean_signal + np.random.normal(0, noise_lvl, len(t))

return results

data_7T = simulate_system(base_7T, E_max_7T, noise_7T)

data_14T = simulate_system(base_14T, E_max_14T, noise_14T)

\# ==========================================

\# 4. PLOTTING

\# ==========================================

fig, (ax1, ax2) = plt.subplots(2, 1, figsize=(10, 10))

\# 7T Plot

ax1.axvspan(CNO_onset, CNO_offset, color='gray', alpha=0.1, label='CNO Active')

for met, values in data_7T.items():

ax1.plot(t, values, label=f'{met} (7T)', lw=2)

ax1.set_title("7T fMRS Simulation: Glx & GABA Dynamics")

ax1.set_ylabel("Concentration (a.u.)")

ax1.legend()

\# 14T Plot

ax2.axvspan(CNO_onset, CNO_offset, color='gray', alpha=0.1, label='CNO Active')

for met, values in data_14T.items():

ax2.plot(t, values, label=f'{met} (14T)', lw=2)

ax2.set_title("14T fMRS Simulation: Glu, Gln & GABA Dynamics")

ax2.set_ylabel("Concentration (a.u.)")

ax2.set_xlabel("Time (minutes)")

ax2.legend()

plt.tight_layout()

plt.show()

%% fMRS: SNR Analysis (Before vs After CNO)

clear; clc; close all;

%% 1. Parameters

n = 200; npts = 2048; BW = 4000; B0 = 300.344;

target_ppm = 4.68; lb = 1; npts_zf = 16384;

shift_pts_digital = 65;

avg_window = 9;

t = (0:npts-1)'/BW;

hz_per_ppm = B0;

%% 2. Load Data

myFolder = uigetdir();

if myFolder == 0, return; end

fname_raw = fullfile(myFolder, 'ser');

fid_raw = fopen(fname_raw, 'r');

[DataIn, ~] = fread(fid_raw, [2*npts, n], 'int32');

fclose(fid_raw);

fids = complex(DataIn(1:2:end, :), DataIn(2:2:end, :));

%% 3. Alignment & Dataset-Agnostic Auto-Phasing

f_axis = linspace(-BW/2, BW/2, npts)';

phc1_ramp = exp(1i * 2 * pi * f_axis * (shift_pts_digital / BW));

ppm_scale = linspace(-BW/2, BW/2, npts) / B0 + target_ppm;

fids_corr = zeros(npts, n);

for k = 1:n

spec = fftshift(fft(fids(:,k))) .* phc1_ramp;

fid = ifft(ifftshift(spec));

s_abs = abs(fftshift(fft(fid .* exp(-15*pi*t))));

[~, max_i] = max(s_abs);

df = (target_ppm - ppm_scale(max_i)) * B0;

fid = fid .* exp(1i * (2 * pi * df * t));

temp_spec = fftshift(fft(fid .* exp(-lb*pi*t)));

[~, naa_idx] = min(abs(ppm_scale - 2.01));

anchor_phase = angle(temp_spec(naa_idx));

fids_corr(:,k) = fid * exp(-1i * anchor_phase);

end

%% 4. SNR & Block Analysis

% Define Block 1 (Before) and Block 2 (After)

fid_blocks = {1:60, 120:200};

snr_values = zeros(length(fid_blocks), 1);

ppm_zf = linspace(-BW/2, BW/2, npts_zf) / B0 + target_ppm;

figure('Color', 'w', 'Position', [100, 100, 1000, 500]);

for b = 1:length(fid_blocks)

% 1. Average the FIDs in the block

avg_fid = mean(fids_corr(:, fid_blocks{b}), 2) .* exp(-lb*pi*t);

spec_raw = real(fftshift(fft(avg_fid, npts_zf)));

% 2. Signal: NAA Peak Amplitude (2.01 ppm)

sig_mask = ppm_zf > 1.9 & ppm_zf < 2.1;

naa_amp = max(spec_raw(sig_mask));

% 3. Noise: SD of baseline (5.0 to 8.0 ppm)

noise_mask = ppm_zf > 5.0 & ppm_zf < 8.0;

noise_floor = spec_raw(noise_mask);

noise_sd = std(detrend(noise_floor)); % Detrend to remove baseline drift

% 4. Absolute SNR Calculation

snr_values(b) = naa_amp / noise_sd;

% Plotting for Visual Verification

subplot(1, 2, b)

plot(ppm_zf, spec_raw, 'k'); hold on;

fill([5 8 8 5], [min(ylim) min(ylim) max(ylim) max(ylim)], 'y', 'FaceAlpha', 0.1, 'EdgeColor', 'none');

set(gca, 'XDir', 'reverse', 'Box', 'off'); xlim([0.5 8.5]);

title(sprintf('Block %d (SNR = %.2f)', b, snr_values(b)));

xlabel('ppm'); ylabel('Intensity');

if b == 1, text(6.5, max(ylim)*0.8, 'Noise Region', 'Color', 'k', 'FontWeight', 'bold'); end

end

%% 5. Terminal Summary

avg_session_snr = mean(snr_values);

fprintf('\n================ SNR SUMMARY ================\n');

fprintf('SNR Before CNO (Block 1): %.2f\n', snr_values(1));

fprintf('SNR After CNO (Block 2): %.2f\n', snr_values(2));

fprintf('---------------------------------------------\n');

fprintf('AVERAGE SESSION SNR: %.2f\n', avg_session_snr);

fprintf('=============================================\n');
